# Integrin alpha5 in human breast cancer is a mediator of bone metastasis and a therapeutic target for the treatment of osteolytic lesions

**DOI:** 10.1038/s41388-020-01603-6

**Published:** 2021-01-08

**Authors:** Francesco Pantano, Martine Croset, Keltouma Driouch, Natalia Bednarz-Knoll, Michele Iuliani, Giulia Ribelli, Edith Bonnelye, Harriet Wikman, Sandra Geraci, Florian Bonin, Sonia Simonetti, Bruno Vincenzi, Saw See Hong, Sofia Sousa, Klaus Pantel, Giuseppe Tonini, Daniele Santini, Philippe Clézardin

**Affiliations:** 1 INSERM, UMR_S1033, LYOS, Lyon, France; 2 Univ Lyon, Villeurbanne, France; 3 Medical Oncology Department, Campus Bio-Medico University of Rome, Rome, Italy; 4 Institut Curie, Service de Génétique, Unité de Pharmacogénomique, Paris, France; 5 Department of Tumor Biology, University Medical Centre Hamburg-Eppendorf, Hamburg, Germany; 6 Laboratory of Translational Oncology, Medical University of Gdansk, Gdansk, Poland; 7 INRA, UMR-754, Lyon, France; 8 Oncology and Metabolism Department, University of Sheffield, Sheffield, UK

**Keywords:** Bone metastases, Integrins

## Abstract

Bone metastasis remains a major cause of mortality and morbidity in breast cancer. Therefore, there is an urgent need to better select high-risk patients in order to adapt patient’s treatment and prevent bone recurrence. Here, we found that integrin alpha5 (ITGA5) was highly expressed in bone metastases, compared to lung, liver, or brain metastases. High *ITGA5* expression in primary tumors correlated with the presence of disseminated tumor cells in bone marrow aspirates from early stage breast cancer patients (*n* = 268; *p* = 0.039). *ITGA5* was also predictive of poor bone metastasis-free survival in two separate clinical data sets (*n* = 855, HR = 1.36, *p* = 0.018 and *n* = 427, HR = 1.62, *p* = 0.024). This prognostic value remained significant in multivariate analysis (*p* = 0.028). Experimentally, *ITGA5* silencing impaired tumor cell adhesion to fibronectin, migration, and survival. *ITGA5* silencing also reduced tumor cell colonization of the bone marrow and formation of osteolytic lesions in vivo. Conversely, *ITGA5* overexpression promoted bone metastasis. Pharmacological inhibition of ITGA5 with humanized monoclonal antibody M200 (volociximab) recapitulated inhibitory effects of *ITGA5* silencing on tumor cell functions in vitro and tumor cell colonization of the bone marrow in vivo. M200 also markedly reduced tumor outgrowth in experimental models of bone metastasis or tumorigenesis, and blunted cancer-associated bone destruction. ITGA5 was not only expressed by tumor cells but also osteoclasts. In this respect, M200 decreased human osteoclast-mediated bone resorption in vitro. Overall, this study identifies ITGA5 as a mediator of breast-to-bone metastasis and raises the possibility that volociximab/M200 could be repurposed for the treatment of ITGA5-positive breast cancer patients with bone metastases.

## Introduction

Breast cancer can be successfully treated when the disease is detected early, but the patient survival markedly decreases once metastatic spread occurs [1]. In this respect, the prognosis for patients with bone metastasis is generally poor and accompanied by skeletal complications (pathological fractures, bone pain, disability) [2]. Several studies have underlined that tumor cell dissemination to the bone marrow is an early metastasis event and represents an independent prognostic factor for poor clinical outcome [3–5]. The bone marrow acts as a reservoir where disseminated tumor cells (DTCs) could survive in a cell-cycle arrest state for long periods of time until environmental conditions are sufficiently permissive for proliferation, at which time they become competent to seed secondary organs and/or cause overt local bone metastasis [6–8]. Molecular mechanisms regulating bone homing and colonization by breast cancer cells remain, however, still poorly understood.

In this study, we searched for potential target genes involved in breast cancer dissemination to distant organs using in silico transcriptomic analyses of primary tumors and metastases. We found that integrin alpha5 (ITGA5) is expressed at high levels in bone metastases compared to non-bone metastases. Furthermore, multivariate analysis showed that *ITGA5* expression in primary breast tumors is an independent prognostic factor for bone relapse. ITGA5 heterodimerizes with integrin beta1 to form the fibronectin receptor α5β1 [9]. In breast cancer, ITGA5 mediates tumor cell adhesion, extracellular matrix-guided directional migration along fibronectin, and tumor cell survival in vitro [9–13]. ITGA5 also mediates lung metastasis in animal models of breast cancer [14, 15]. Additionally, a synthetic peptide inhibitor derived from the synergy region of fibronectin that binds to α5β1 and αvβ3 integrins (ATN-161, also called PHSCN) reduces both MDA-MB-231 breast cancer bone metastasis formation and skeletal tumor outgrowth [14, 16]. However, ATN-161 interacts with αvβ3 [16], and the treatment of tumor-bearing animals with a specific nonpeptide antagonist of αvβ3 (PSK 1404) also inhibits bone metastasis formation [17], suggesting that the inhibitory effect of ATN-161 on bone metastasis formation was mediated through the therapeutic targeting of αvβ3. Besides ATN-161, a humanized IgG4 monoclonal antibody against α5β1, known as M200 (volociximab), was developed as an antiangiogenic agent for the treatment of solid tumors and age-related macular degeneration [18, 19]. A phase I study conducted in 22 patients with advanced stage solid tumors showed that the pharmaco-toxicologic profile of M200 is safe, and preliminary evidence of antitumor activity was reported in one patient with renal cell carcinoma [18]. Clinical trials also evaluated its safety in the treatment of ovarian cancer and non-small cell lung cancer, as a single agent or in combination with chemotherapy [20, 21].

Here, we provide evidence that ITGA5 is a mediator of bone metastasis and a potential therapeutic target for bone metastasis treatment. Using genetic overexpression or silencing strategies, we show that ITGA5 in breast cancer cells mediates metastatic tumor cell colonization of the bone marrow and promotes formation of osteolytic lesions in vivo. Furthermore, we show that M200 could be effective in the treatment of breast cancer patients with osteolytic bone metastases by targeting both tumor cells and osteoclasts, the latter being bone-resorbing cells that mediate cancer-induced bone destruction.

## Results

### ITGA5 is a bone metastasis-associated gene in breast cancer

**Fig. 1:**
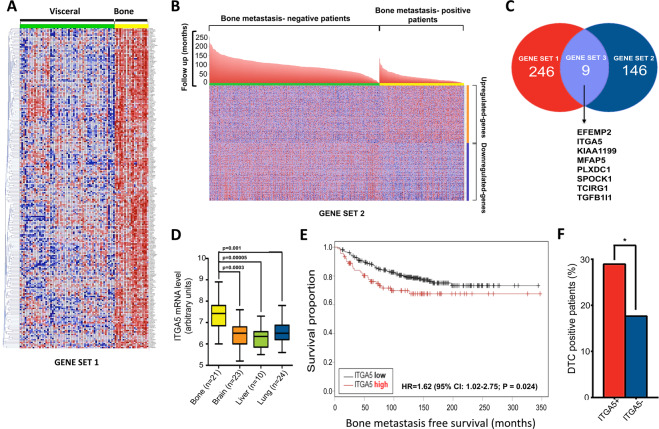
ITGA5 is a bone metastasis-associated gene in breast cancer. **A** Heat map analysis of genes that are highly expressed in bone metastases (*n* = 21) compared to visceral metastases (*n* = 59). Each row represents a gene, and each column represents a metastasis specimen. Class comparison analysis was performed using a univariate *t*-test (*p* < 10^−4^, fold-change > 1.5). **B** Heat map of genes associated with bone metastasis-free survival. Transcriptomic gene profile of 855 radically resected primary breast tumors was hierarchized according to the location of the first distant metastasis. Bone metastasis-negative patients: patients who first relapsed at non-bone sites or did not relapse after 200 months follow-up. Bone metastasis-positive patients: patients who first relapsed in bone after 100 months follow-up. **C** Venn diagram of (*a*) genes upregulated in bone vs. visceral breast cancer metastasis (gene set #1) and (*b*) genes upregulated in primary tumors from patients with early occurrence of bone metastasis (gene set #2), which underscores a set of eight genes (gene set #3) shared between gene sets #1 and #2: *EFEMP2* EGF-containing fibulin-like extracellular matrix protein 2, *ITGA5* integrin alpha5, *KIAA1199* cell migration-inducing and hyaluronan-binding protein (CEMIP), *MFAP5* microfibrillar-associated protein 5, *PLXDC1* plexin domain-containing protein 1, *SPOCK1* SPARC (osteonectin), Cwcv and kazal-like domains proteoglycan 1, *TCIRG1* T-cell immune regulator 1, *TGFB1l1* transforming growth factor beta1-induced transcript 1. **D** *ITGA5* mRNA expression levels in breast cancer metastases. Data are expressed as mean ± SEM. **E** Kaplan–Meier estimates for rates of bone metastasis-free survival of breast cancer patients (*n* = 427), according to high and low *ITGA5* expression levels. HR hazard ratio, CI confident interval. HR and 95% CI are based on Cox univariate analysis. **F** Percentage of breast cancer patients with DTCs in the bone marrow according to high or low *ITGA5* expression levels in matched primary mammary tumors (*n* = 268). ITGA5+ ITGA5-high, ITGA5− ITGA5-low. *p = 0.039.

We compared the transcriptomic profile of 21 bone metastases with that of 59 metastases from other distant organs. This analysis identified 246 genes (gene set #1) that were expressed at higher levels in bone metastases compared to non-bone metastases (Fig. 1A and Table S1). In parallel, the analysis of 855 radically resected primary breast tumors with known location of the first distant metastasis led to 146 genes (gene set #2) that were significantly upregulated in primary tumors from patients who first relapsed in bone, compared to patients who first relapsed at non-bone metastatic sites or did not relapse after 200 months follow-up (Fig. 1B and Table S1). Eight genes were common to gene sets #1 and #2: EGF-containing fibulin-like extracellular matrix protein 2 (*EFEMP2*), *ITGA5*, *KIAA1199* (cell migration-inducing and hyaluronan-binding protein), microfibrillar-associated protein 5 (*MFAP5*), plexin domain-containing protein 1 (*PLXDC1*), SPARC (Osteonectin), Cwcv and kazal-like domains proteoglycan 1 (*SPOCK1*), T-cell immune regulator 1 (*TCIRG1*), and transforming growth factor beta1-induced transcript 1 (*TGFB1I1*) (Fig. 1C). Besides the role played by ITGA5 in promoting breast cancer cell adhesion, invasion, and survival [9–16], EFEMP2, KIAA1199, and MFAP5 also enhance breast cancer motility and invasiveness [22–24]. SPOCK1 and TGFB1I1 (also called hydrogen peroxide-inducible clone 5) are induced by TGF-β and promote breast cancer cell invasion [25, 26]. PLXDC1 increases invasion in gastric cancer [27], and TCIRG1 is an osteoclast-specific vacuolar proton pump subunit that acts as a metastasis enhancer in hepatocellular carcinoma [27]. In addition, MFAP5 is upregulated in human breast cancer bone metastases compared to primary tumors [24].

The functional importance of these genes was assessed by gene network analysis, revealing a prominent role for *ITGA5*, given its high connectivity degree within the network structure (Fig. S1). Moreover, as shown in Fig. 1D, *ITGA5* was highly expressed in bone metastases compared to lung (*p* = 0.001), liver (*p* = 5.10^−5^), and brain (*p* = 3.10^−4^) metastases. We therefore focused our attention to the role of ITGA5 in breast cancer bone metastasis.

### ITGA5 is an independent prognostic factor for breast cancer bone metastasis

We quantified *ITGA5* expression levels in 427 radically resected primary breast tumors [28]. Kaplan–Meier survival analysis revealed that the risk of bone metastasis was significantly higher for patients with high ITGA5 levels (HR = 1.62, *p* = 0.024) (Fig. 1E). Furthermore, ITGA5 predicted bone relapse (*p* = 0.028) independently of clinicopathological characteristics (Table 1). To confirm these findings, we conducted in silico analysis of a cohort of 855 radically resected primary mammary tumors with clinical annotation for recurrences and observed that breast cancer patients with tumors expressing high *ITGA5* mRNA levels were more likely to relapse in bone (HR = 1.36, *p* = 0.018) (Fig. S2). After adjusting for clinicopathological factors, *ITGA5* remained significantly associated with bone relapse (*p* = 0.034) (Fig. S2).

**Table 1:** Association of ITGA5 expression with clinical and biological characteristics of patients with early stage breast cancer from the Curie Institute/Centre René Huguenin cohort^a^.

Characteristics	Hazard ratio	95% CI lower limit	95% CI upper limit	*p* value
Age	1.000	0.985	1.016	0.965
Tumor size	1.003	0.991	1.015	0.650
Nodal status	2.635	1.271	5.462	**0.009**
Estrogen receptor status	0.825	0.524	1.297	0.404
Progesterone receptor status	1.267	0.858	1.872	0.234
Her2 status	0.784	0.526	1.170	0.234
ITGA5 expression	1.359	1.083	1.707	**0.028**

^a^Hazard ratios and 95% confidence intervals (CIs) are based on Cox multivariate regression analysis.

Bold value highlight statistically significant characteristics.

### Elevated ITGA5 protein levels in primary tumors are associated with the presence of DTCs in bone marrow aspirates from patients with breast cancer

To examine the potential contribution of ITGA5 in the homing of breast cancer cells to bone, we analyzed by immunohistochemistry ITGA5 protein levels in 268 radically resected primary tumors from a cohort of breast cancer patients with no clinical signs of metastasis for whom the presence or absence of DTCs in the bone marrow was documented (Table S2) [29]. A significantly higher percentage of breast cancer patients having elevated ITGA5 protein levels in primary tumors were DTC-positive (*p* = 0.039), compared to that observed for patients with low ITGA5 levels in primary tumors (Fig. 1F and Table S2). Additionally, flow cytometry analysis of a breast cancer DTC cell line (BC-M1) [30, 31] showed cell surface expression of integrin α5β1 (Fig. S3A).

### ITGA5 promotes breast cancer cell dissemination to the bone marrow and formation of osteolytic bone metastases in vivo

Human MDA-MB-231, Hs578T, and MDA-B02 breast cancer cells, which are ER- and PR-negative and do not bear an amplification of HER2 gene (referred to as triple-negative breast cancer cells), had higher cell surface expression levels of integrin α5β1 and higher ITGA5 protein levels than luminal A (T47D, MCF-7, BT-474) and HER2-expressing luminal B (SKBr3) breast cancer cell lines, as judged by flow cytometry and western blotting, respectively (Fig. S3A, B). *ITGA5* mRNA expression levels in tumor cells were further investigated using 51 distinct breast cancer cell lines with different molecular phenotypes and degree of invasiveness (GSE12777) [32]. A significant correlation was observed between high *ITGA5* mRNA expression levels and high tumor cell invasiveness (*p* = 0.0083) (Fig. S3C, D). In particular, the highest *ITGA5* mRNA levels were observed in claudin-low, triple-negative breast cancer cell lines (*p* < 0.01) (Fig. S3E).

**Fig. 2:**
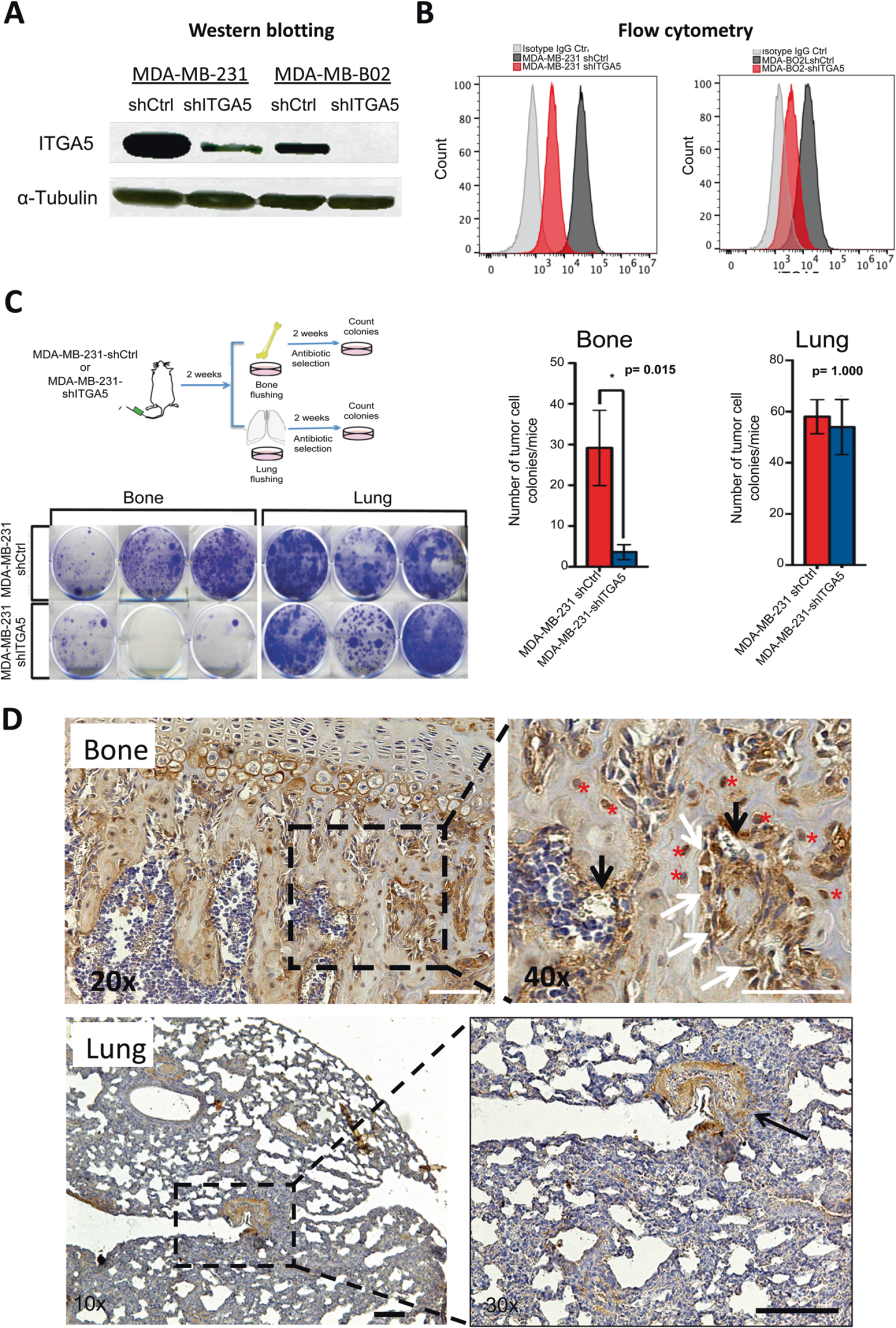
ITGA5 in triple-negative MDA-MB-231 and MDA-B02 breast cancer cells promotes the burden of micrometastatic disease in the bone marrow in vivo. **A** ITGA5 expression in MDA-MB-231 and MDA-B02 cells silenced for ITGA5 (shITGA5), compared to control cell lines (shCtrl), as measured by western blotting. **B** Cell surface expression levels of integrin α5β1 in MDA-MB-231 (left-hand panel) and MDA-B02-shCtrl and shITGA5 cells (right-hand panel), as measured by flow cytometry using anti-ITGA5 monoclonal antibody IIA1 (red and black histograms) or an isotype-matched negative control antibody (gray histograms). **C** Top-left panel: schematic representation of the experimental protocol. MDA-MB-231-shCtrl or MDA-MB-231-shITGA5 cells were inoculated intra-arterially to Balb/c *nude* mice (*n* = 5 per group). Two weeks after tumor cell inoculation, animals were culled, and the bone marrow and lungs collected for tumor cell colony assays. Bottom-left panel: representative images of tumor cell colonies in the bone marrow and lung are shown for each cell line. Right panel: bar graphs showing the average number of tumor cell colonies formed in the bone marrow and lungs for each cell line. Data are expressed as the mean ± SEM. **D** Fibronectin immunostaining in bone and lung. Right-hand panels are a magnification of insets shown in left-hand panels. In bone, a strong immunostaining for fibronectin was observed in osteoblasts (white arrows), osteocytes (red asterisks), and endothelial cells (black arrows). In lungs, the immunostaining was mainly localized around blood vessels.

We therefore silenced ITGA5 in claudin-low MDA-MB-231 and MDA-B02 breast cancer cells, the latter being a bone metastatic cell subpopulation of the MDA-MB-231 cell line, which constitutively and specifically overexpresses αvβ3 integrin compared to the parental cell line [17, 33]. ShRNA-mediated silencing of *ITGA5* in these cells drastically reduced ITGA5 expression, both at the protein and cell surface expression levels compared to shRNA control cells (Fig. 2A, B). The flow cytometry analysis of shITGA5-MDA-MB-231 and shITGA5-MDA-B02 cells showed that the silencing of ITGA5 did not modify cell surface expression levels of integrin subunits α2, α3, α4, and β1 and of αvβ3 integrin, when compared to shRNA control cells (Figs. S4 and S5). The silencing of ITGA5 led to a 60% reduction of tumor cell adhesion to fibronectin (Fig. S6A), whereas tumor cell adhesion to glass, poly-D-Lysine, and laminin remained unchanged (Fig. S6B). In addition, *ITGA5* silencing reduced by half the number and size of mammospheres formed by MDA-B02-shITGA5 cells, compared to that observed with MDA-B02-shCtrl cells (Fig. S6C).

To investigate whether ITGA5 could drive tumor cell anchorage in bone marrow in vivo, MDA-MB-231 cells that have the propensity to form lung and bone metastases were injected into the tail artery of immunodeficient mice and, 2 weeks after tumor cell inoculation, these animals were culled and the number of micrometastases in bone marrow and lungs quantified. Abrogating *ITGA5* expression in MDA-MB-231-ShITGA5 cells significantly reduced bone marrow micrometastasis formation (*p* = 0.015), whereas the extent of tumor cell dissemination to lungs remained unchanged, compared to MDA-MB-231-Sh-Ctrl cells (Fig. 2C). These results may be explained by the fact that fibronectin was strongly expressed in bone tissue, whereas only a weak expression was observed in lungs, the immunostaining being essentially localized around blood vessels within the lung parenchyma (Fig. 2D). Moreover, high fibronectin expression levels in bone marrow stroma were observed compared to lung parenchyma, when analyzing EST profiles of *Mus Musculu*s and *Homo Sapiens* tissue samples (Fig. S7). Thus, these data suggested that ITGA5 preferentially mediates tumor cell anchorage in the bone marrow by binding to fibronectin.

To determine whether ITGA5 could play a role in the formation and progression of bone metastases, bone-seeking MDA-B02 cells, silenced or not silenced for *ITGA5*, were injected into the tail artery of immunodeficient mice. Radiographic analysis of tumor-bearing animals 4 weeks after tumor cell inoculation showed a significant decrease (*p* = 0.0268) of the extent of osteolytic lesions in hind limbs of mice injected with MDA-B02-shITGA5 cells compared to that observed with MDA-B02-shCtrl cells (Fig. S8).

**Fig. 3:**
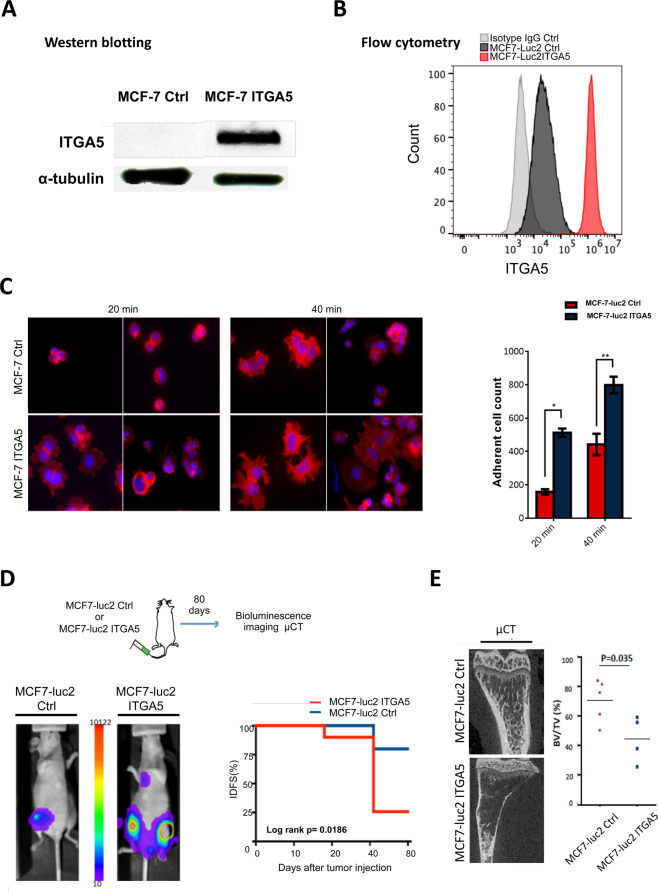
Overexpression of ITGA5 in human luminal A MCF-7 breast cancer cells promotes tumor cell adhesion to fibronectin in vitro and enhances skeletal tumor burden and the extent of metastatic osteolytic lesions in vivo. **A** Western blot analysis of ITGA5 in MCF-7-luc2 cells (MCF-7 Ctrl) after transduction with the retroviral plasmid (MCF-7 ITGA5). **B** Flow cytometry analysis of cell surface expression of α5β1 integrin in MCF-7-Ctrl and MCF-7-ITGA5 cells. **C** Left panels: representative images of MCF-7-Ctrl and MCF-7-ITGA5 cell adhesion to fibronectin as a function of time. Right panel: quantification of the number of adherent cells to fibronectin at 20 and 40 min. *p < 0.001; **p < 0.0001. **D** Top panel: schematic representation of the experimental protocol. MCF-7-luc2-Ctrl or MCF-7-luc2-ITGA5 cells were inoculated intra-arterially to Balb/c *nude* mice (*n* = 4–5 per group). Eighty days after tumor cell inoculation, animals were analyzed by bioluminescence imaging, and histomorphometry of metastatic legs was measured by microcomputed tomography (μCT). Bottom-left panel: whole-body bioluminescence imaging of a representative animal for each group at day 80 after tumor cell inoculation. Bottom-right panel: Kaplan–Meier estimates for rates of invasive-disease-free survival (IDFS) of animals, as measured by bioluminescence imaging. **E** Left panel: μCT of representative metastatic tibiae for each group. Right panel: Assessment of bone destruction by histomorphometry, as measured by the bone volume (BV)/tissue volume (TV) ratio of metastatic legs from mice injected with MCF-7-luc2-Ctrl (*n* = 5) or MCF-7-luc2-ITGA5 cells (*n* = 4).

Experiments were also conducted with human MCF-7 breast cancer cells that express low amounts of ITGA5 (Fig. S3A, B). Transduction of luciferase2-expressing MCF-7 cells (MCF-7-luc2) with a retroviral plasmid containing the ITGA5 open reading frame (MCF-7-luc2-ITGA5) resulted in a strong expression of integrin α5β1 (Fig. 3A, B). As judged by flow cytometry analysis, ITGA5 overexpression led to decreased cell surface expression levels of α2 and α3 integrins, whereas cell surface expression levels of α4, β1, and αvβ3 remained unchanged, when compared to control MCF-7-luc2 cells (Figs. S4 and S5). Integrins α2β1 and α3β1 are acting as cell surface receptors for collagen and laminin, respectively [34]. We cannot exclude a decreased attachment of MCF-7-luc2-ITGA5 cells to these extracellular matrix proteins. However, as expected, MCF-7-luc2-ITGA5 cell adhesion and spreading to fibronectin were increased compared to that observed with MCF-7-luc2-Ctrl cells (Fig. 3C). MCF-7-luc2-ITGA5 or MCF-7-luc2-Ctrl cells were therefore inoculated intra-arterially to *nude* mice (Fig. 3D, E). Bioluminescence imaging revealed an earlier onset (*p* = 0.0186) of skeletal tumor burden in mice injected with MCF-7-luc2-ITGA5 cells, compared to animals bearing MCF-7-luc2-Ctrl cells (Fig. 3D). Microcomputed tomography of metastatic long bones showed that the BV/TV ratio (a measure of the bone volume) was decreased (*p* = 0.035) in mice inoculated with MCF-luc2-ITGA5 cells, indicating a higher extent of bone destruction compared to animals bearing MCF-7-luc2-Ctrl tumor cells (Fig. 3E).

Overall, these data indicated that ITGA5 mediates the homing of breast cancer cells in the bone marrow and promotes formation of osteolytic bone metastases in vivo.

### Pharmacological inhibition of ITGA5 reduces breast cancer cell dissemination to the bone marrow and formation of osteolytic bone metastases in vivo

**Fig. 4:**
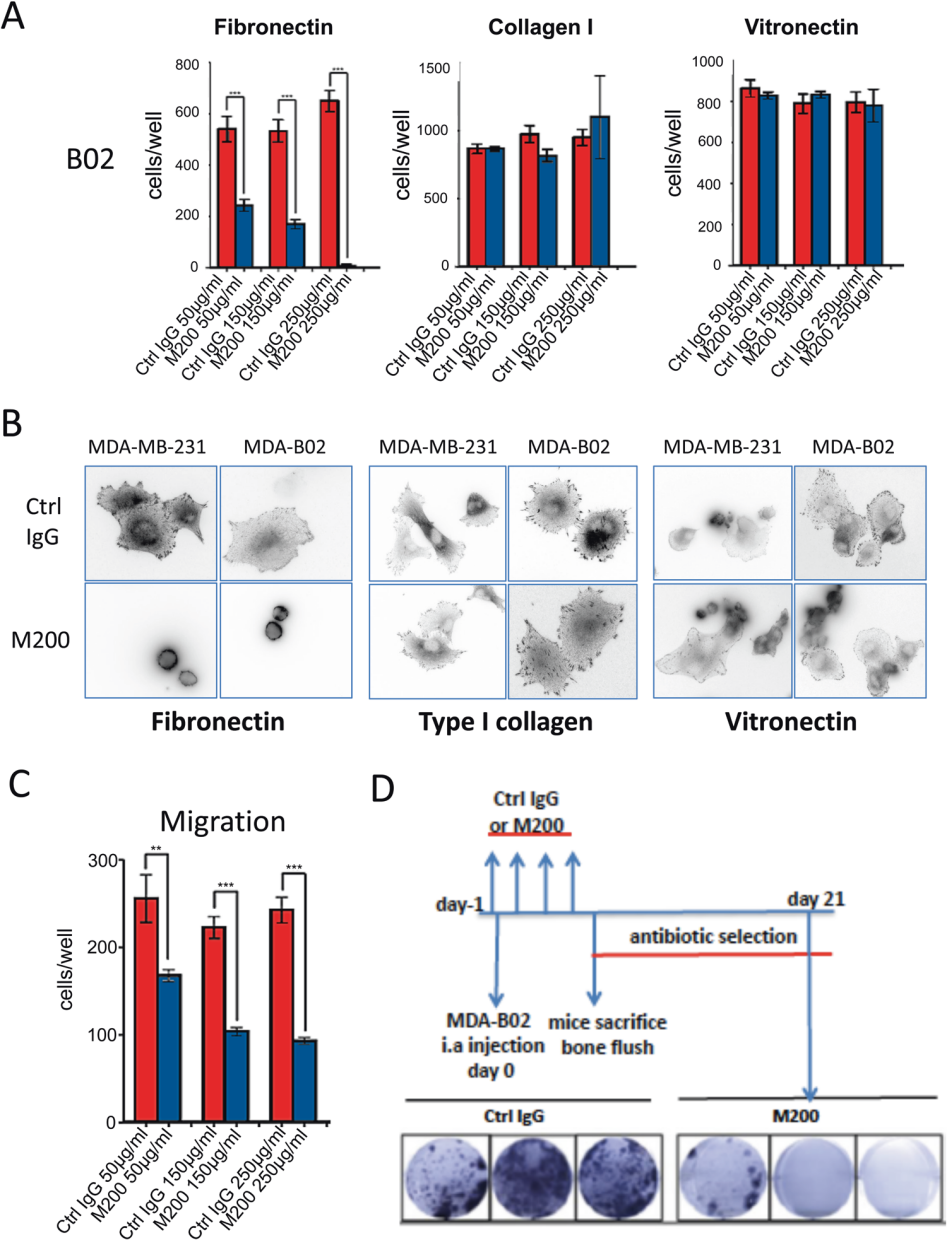
Function-blocking humanized anti-ITGA5 monoclonal antibody M200 specifically inhibits breast cancer cell adhesion and spreading to fibronectin and blocks MDA-B02 cell colonization in the bone marrow. **A** MDA-B02 cells treated or not treated with increasing concentrations of M200 (50, 150, and 250 μg/ml) were allowed to adhere for 1 h to human fibronectin, type I collagen, or vitronectin. Attached cells were then fixed, stained, and counted under microscope. Data are expressed as mean ± SEM of three separate experiments. ***p < 0.0001. **B** Representative images of paxillin immunofluorescent labeling of focal adhesion contacts (black spots at the edge of the plasma membrane) in MDA-MB-231 and MDA-B02 cells treated with a control IgG or M200 (250 μg/ml) that attached and spread to fibronectin, type I collagen, or vitronectin. **C** Effect of increasing concentrations of control IgG or M200 (50, 150, and 250 μg/ml) on MDA-B02 cell migration through 8-µm diameter pore-size inserts coated with fibronectin. Data are the mean ± SEM of three separate experiments. **p < 0.001; ***p < 0.0001. **D** Top-left panel: schematic representation of the treatment protocol. MDA-B02 cells were inoculated intra-arterially to Balb/c *nude* mice. Animals received a treatment with a control IgG or M200 (15 mg/kg) every other day, starting 1 day before tumor cell inoculation. Seven days after tumor cell inoculation, animals were culled, and the bone marrow collected for tumor cell colony assays. Bottom panel: representative images of tumor cell colonies in the bone marrow from animals treated with M200 or the control IgG.

**Fig. 5:**
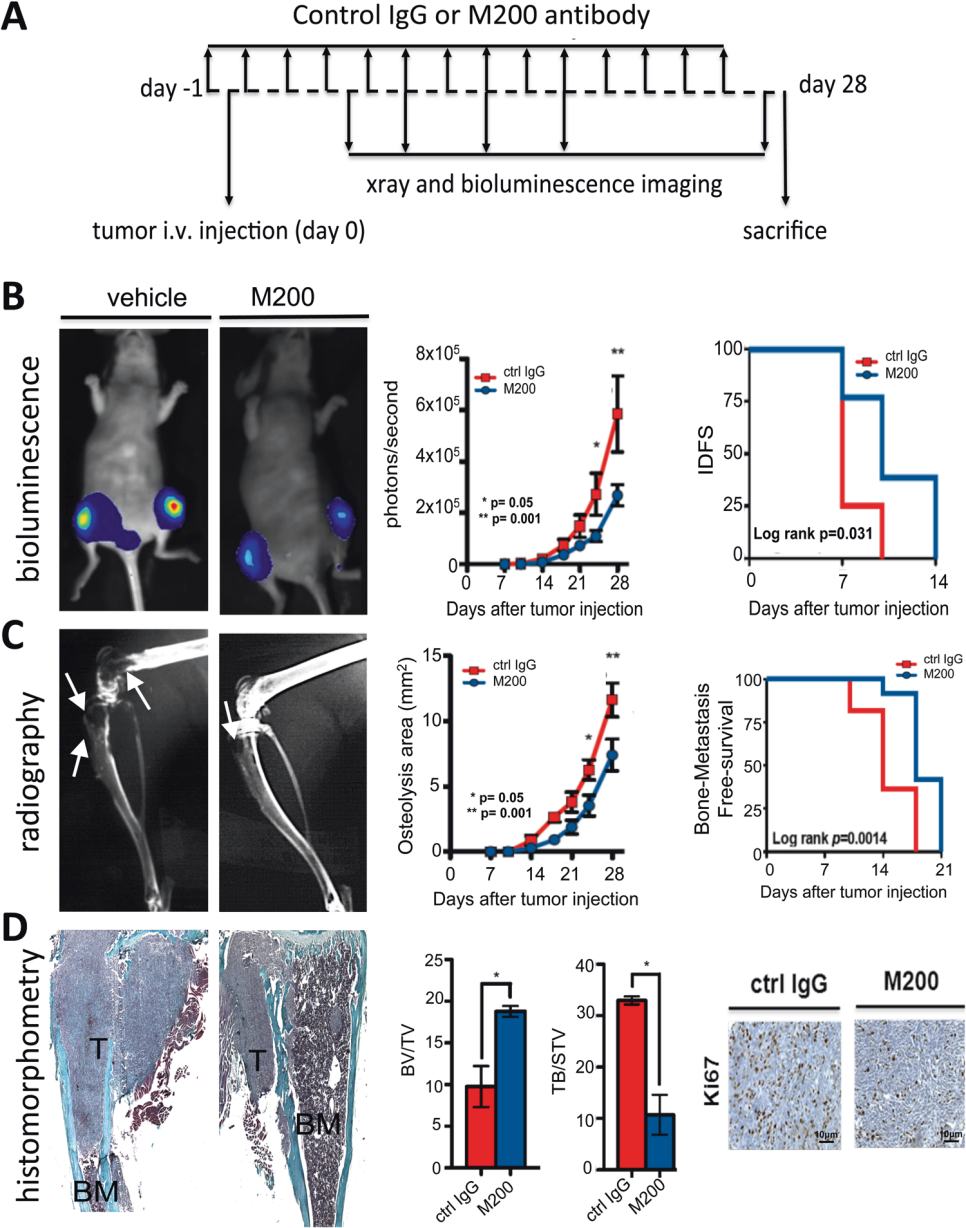
Pharmacological inhibition of ITGA5 with M200 treatment reduces bone metastasis formation in vivo. **A** Top panel: schematic representation of the treatment protocol. MDA-B02 cells were inoculated intra-arterially to Balb/c *nude* mice. Animals (ten mice per group) received a treatment with a control IgG or M200 (15 mg/kg) every other day, starting 1 day before tumor cell inoculation. Bone metastasis formation in animals was monitored over time by bioluminescence imaging and radiography. Twenty-eight days after tumor cell inoculation, animals were culled, and metastatic bones were analyzed by histomorphometry and immunohistochemistry. **B** Left panels: whole-body bioluminescence imaging of a representative animal for each group at day 28. Middle panel: progression of tumor burden in control IgG- and M200-treated animals, as measured by whole-body bioluminescence imaging. Right panel: Kaplan–Meier estimates for rates of invasive-disease-free survival (IDFS) of animals, as measured by bioluminescence imaging. **C** Left panels: radiograph of a representative metastatic leg for each group at day 28. Middle panel: progression of osteolytic lesion areas in control IgG- and M200-treated animals, as measured by radiography. Right panel: Kaplan–Meier estimates for rates of bone metastasis-free survival (BMFS) of animals, as measured by radiography. **D** Left panels: Goldner’s trichrome staining of tibial tissue sections of metastatic legs from tumor-bearing mice treated with the control IgG or M200. Bone is stained green, whereas bone marrow (BM) and tumor cells are stained purple. Middle panel: assessment of bone destruction and tumor burden as measured, respectively, by the bone volume (BV)/tissue volume (TV) ratio and tumor burden (TB)/soft tissue volume (STV) ratio of metastatic legs from tumor-bearing mice treated with the control IgG or M200. *P < 0.05. Right panel: representative Ki67 staining of tumor areas in bone tissue sections from metastatic legs of animals treated with the control IgG or M200.

We examined the therapeutic potential of targeting ITGA5 for the treatment of bone metastasis, using a humanized monoclonal antibody against α5β1 (M200, volociximab) [18]. Antibody M200 selectively binds to human α5β1, but not murine α5β1 [18]. In vitro, M200 treatment dose-dependently decreased MDA-B02 cell adhesion to fibronectin (*p* < 0.0003), but not to type I collagen, or vitronectin (Fig. 4A). The paxillin immunofluorescent labeling of focal adhesion contacts showed that M200 specifically inhibited MDA-B02 and MDA-MB-231 cell spreading to a fibronectin matrix, but not to type I collagen or vitronectin (Fig. 4B). In line with this inhibitory effect on tumor cell spreading, M200 treatment dose-dependently reduced MDA-B02 cell migration through inserts coated with fibronectin (*p* < 0.001) (Fig. 4C). In vivo, immunodeficient mice were treated with M200 or a negative control IgG antibody beginning 1 day (D1) before intra-arterial inoculation of MDA-B02 cells (D0). The treatment with the antibody then continued every other day until day 7, at which time animals were culled, and the bone marrow collected and placed under antibiotic selection, enabling the selective outgrowth of antibiotic-resistant tumor cells (Fig. 4D). After 2 weeks in culture, the average number of tumor cell colonies recovered in the bone marrow from animals treated with M200 was significantly impaired, compared to that recovered from animals treated with a control IgG (7 ± 2 and 128 ± 10 colonies/well, respectively; *p* < 0.0003) (Fig. 4D). Using a similar treatment protocol from D1 to D28 (Fig. 5A), antibody M200 also significantly delayed the onset of skeletal tumor burden and extent of osteolytic lesions in animals (Fig. 5B, C). Histomorphometric analysis of metastatic legs from M200-treated animals showed that the BV/TV ratio was enhanced when compared with control IgG-treated tumor-bearing animals (Fig. 5D). This difference was accompanied by a sharp reduction in the TB/STV ratio (a measure of the skeletal tumor burden) (Fig. 5D). Moreover, immunostaining of metastatic legs showed a concomitant decrease of Ki67 index, a measure of tumor cell proliferation (M200, 15.5 ± 2.5% vs. Ctrl IgG, 28.5 ± 2.5%; *p* < 0.05) (Fig. 5D).

**Fig. 6:**
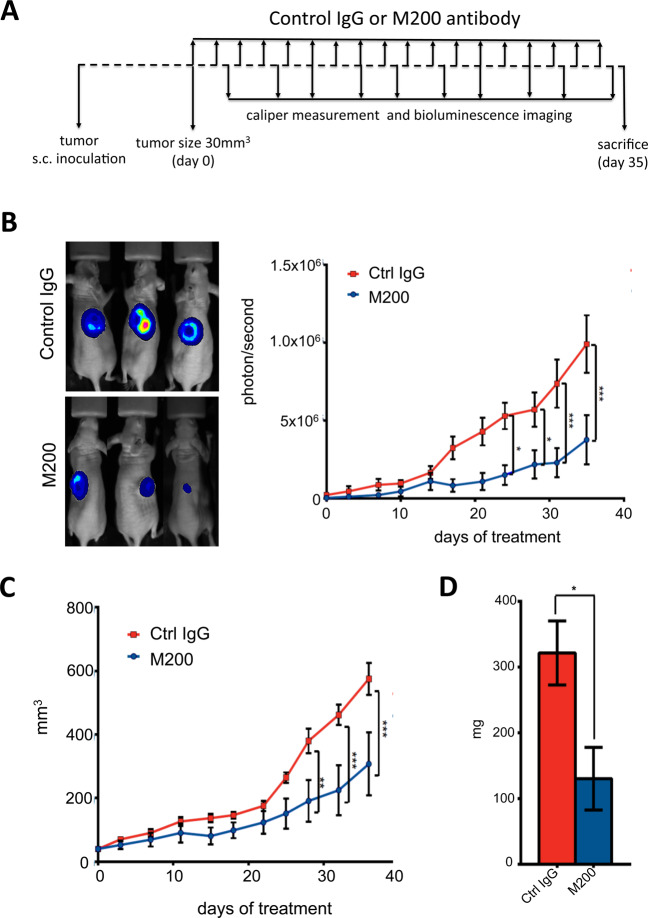
Pharmacological inhibition of ITGA5 with M200 treatment reduces growth of subcutaneous tumor xenografts in vivo. **A** Schematic representation of the treatment protocol. MDA-B02 cells were inoculated subcutaneously to Balb/c *nude* mice. Animals (five mice per group) received a treatment with a control IgG or M200 (15 mg/kg) every other day, starting 1 day before tumor cell inoculation. Tumor growth in animals was monitored over time by bioluminescence imaging and using a vernier caliper. At day 35 after tumor cell inoculation animals were culled and tumors weighted. **B** Left panel: whole-body bioluminescence imaging of three representative mice per group at day 35. Right panel: tumor burden curves of tumor-bearing animals treated with the control IgG or M200, as judged by bioluminescence imaging (photons/second). **C** Tumor growth curves of tumor-bearing animals treated with the control IgG or M200, as judged by vernier caliper measurement (mm^3^). **D** Bar graph represents the average weight of tumors for each group. Data are presented as mean ± SEM. *p < 0.05; **p < 0.01; ***p < 0.005.

To be free from the impact of bone-derived growth factors released from resorbed bone that stimulate tumor growth, the antitumor potential of antibody M200 was investigated in animals bearing subcutaneous MDA-B02 tumor xenografts (Fig. 6A). A statistically significant reduction of tumor growth was observed in M200-treated tumor-bearing animals, compared to control IgG-treated tumor-bearing animals (*p* < 0.005) (Fig. 6B, C). At day 35 after tumor cell inoculation, the median weight of tumors from M200-treated animals was almost threefold lower than that of tumors from Ctrl IgG-treated animals (*p* = 0.05) (Fig. 6D). Similarly, ITGA5 silencing in MDA-B02 cells substantially reduced subcutaneous growth of MDA-B02-shITGA5 tumors compared to control (Fig. S9). Furthermore, cell-cycle analysis showed that M200 treatment inhibited MDA-B02 cells entering into S phase, when compared to that observed with a negative control IgG antibody (Fig. S10). Thus, M200 exhibits a direct antitumor effect in vitro and in vivo.

### Anti-ITGA5 function-blocking antibody M200 decreases human osteoclast differentiation and activity

**Fig. 7:**
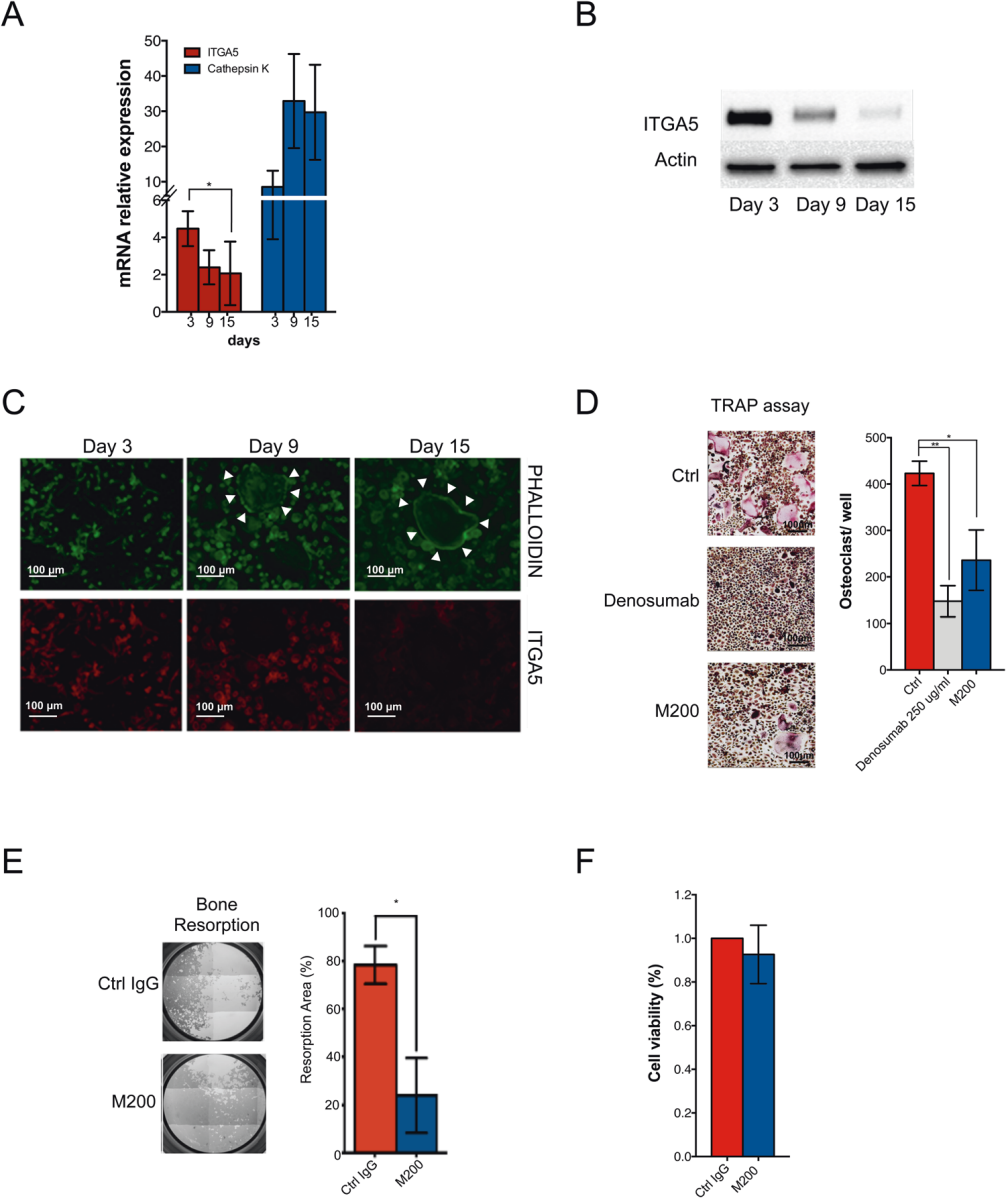
Pharmacological inhibition of ITGA5 with M200 antibody inhibits human osteoclast differentiation and activity in vitro. **A** *ITGA5* and *cathepsin K* mRNA expression levels (relative to GSUb housekeeping gene) at different stages of human osteoclast differentiation. **B** Western blot analysis of ITGA5 at different stages of human osteoclast differentiation. **C** Fluorescence-based staining of ITGA5 and F-actin ring (white arrowheads) at different stages of human osteoclast differentiation, using phycoerythrin-conjugated anti-ITGA5 antibody and FITC-labeled phalloidin, respectively. Scale bar: 100 μm. **D** In vitro osteoclast differentiation of human peripheral blood mononuclear cells treated with M-CSF and RANKL, alone (Ctrl) or in combination with anti-RANKL antibody denosumab (250 μg/ml) or anti-ITGA5 antibody M200 (250 μg/ml). Mature osteoclasts were quantified as multinucleated (more than three nuclei), TRAP-positive cells. Representative images are shown for each group. *,**p < 0.05 and 0.001, respectively. **E** Resorption of an inorganic three-dimensional crystalline material by human osteoclasts treated with a control IgG or M200 (250 μg/ml). Representative images are shown for each group. *p < 0.05. **F** Effect of control IgG and M200 on viability of human osteoclasts, as measured by MTT assay.

M200 did not interfere with murine osteoclastogenesis induced by RANKL and MCS-F in combination with the conditioned medium from MDA-B02 cells (Fig. S11A), nor did it modulate gene expression of murine osteoblast during osteoblastogenesis in vitro (Fig. S11B). However, ITGA5 is expressed in human osteoclasts [35]. We therefore tested the effect of M200 on human osteoclasts, using PBMCs treated with RANKL and MCS-F to induce osteoclast differentiation, as previously described [36]. When compared to cathepsin K (an osteoclast marker), osteoclasts did express ITGA5, both at the mRNA and protein levels (Fig. 7A, B). ITGA5 expression gradually decreased during the course of osteoclast differentiation (Fig. 7B, C). Nevertheless, M200 was nearly as potent as anti-RANKL antibody denosumab to inhibit human osteoclast differentiation in vitro, compared to a control IgG (Fig. 7D). Furthermore, antibody M200 inhibited osteoclast activity, decreasing by 80% the resorption of a synthetic inorganic bone matrix (Fig. 7E). By contrast, it did not affect osteoclast viability (Fig. 7F).

## Discussion

Our study establishes a bone metastasis-promoting role for ITGA5 in breast cancer. Specifically, we report that high *ITGA5* levels in primary tumors were predictive of poor bone metastasis-free survival in two separate clinical data sets (HR = 1.36, *p* = 0.018 and HR = 1.62, *p* = 0.024). Additionally, using a clinical cohort of breast cancer patients without any clinical signs of metastasis, we showed that high *ITGA5* expression levels in primary tumors correlated with the presence of DTCs in the bone marrow. Moreover, ITGA5 was expressed in human DTCs (this study and 12). Our clinical data are consistent with those obtained in previous prospective clinical trials demonstrating that the risk of recurrence in early stage breast cancer is significantly higher in patients with detectable DTCs in the bone marrow than in those without [3–5]. These findings ([3–5], [12], and this study) collectively suggest that ITGA5 mediates DTC colonization of the bone marrow. This contention was also supported by our preclinical data. Using genetic silencing and overexpression strategies or pharmacological inhibition, we uncovered a specific association between ITGA5 expression levels in breast cancer and the development of bone metastasis. Although bone is a predominant site of metastasis for ER-positive breast cancer with a frequency as high as 65–70%, triple-negative breast cancer exhibits a rate of bone metastasis (39%) similar to lung metastasis (43%), which is comparatively higher than that observed in other distant metastatic sites such as brain (25%) and liver (21%) [37]. ITGA5 could therefore contribute to the tropism of triple-negative breast cancer cells to bone. Several factors have been shown to regulate *ITGA5* expression in triple-negative breast cancer [10, 38–40]. For example, steroid receptor coactivator (SRC-1), which is an ER transcriptional coactivator, enhances *ITGA5* expression in ER-negative breast cancer cells [10]. Additional factors expressed by human breast cancer cells, such as PTH-rP and angiopoietin-2, promote tumor cell adhesiveness to fibronectin and tumor cell motility and invasion through the specific upregulation of ITGA5 [38, 39]. Conversely, members of the miR-30 family impede breast cancer bone metastasis formation by directly targeting *ITGA5* [40]. In this respect, *ITGA5* silencing in Hs578T cells (a triple-negative breast cancer cell line expressing high ITGA5 levels) recapitulates inhibitory effects of miR-30s on bone metastasis formation in vivo [40]. Here, we found that abrogating *ITGA5* in human MDA-MB-231 cells also blunted tumor burden in the bone marrow, whereas the formation of pulmonary micrometastases remained unaffected. These results may be explained by the fact that fibronectin is naturally expressed in the bone stroma, whereas its expression in lung parenchyma is essentially localized around blood vessels ([11], [41], and this study). This observation does not preclude a role for ITGA5 in lung metastasis. Indeed, ITGA5 has been associated with lung metastasis in animal models of breast cancer [14, 15]. However, our study suggests that additional molecular mechanisms associated with lung metastasis formation may likely compensate for the lack of ITGA5 in human breast cancer cells, whereas ITGA5 is crucial for the homing of these cancer cells in the bone marrow. It has previously been reported that ITGA5 promotes survival of breast cancer cells in the bone marrow [12]. Here, we showed that ITGA5 silencing reduced the survival of breast cancer cells (Fig. S6C). We therefore propose that ITGA5 provides breast cancer cells (and DTCs) with a survival advantage by binding to fibronectin in the bone marrow, which explains, at least in part, why high ITGA5 expression levels in primary tumors predict the occurrence of future bone metastases in patients with early stage breast cancer.

We previously reported that bone-seeking MDA-B02 cells specifically overexpress αvβ3 integrin, compared to parental MDA-MB-231 cells ([33] and Fig. S5), and its overexpression by MDA-MB-231 cells reproduces the bone metastatic phenotype of MDA-B02 cells in vivo [17]. Here, ITGA5 had a bone metastasis-promoting role, whereas ITGA5 expression in MDA-B02 cells was 20% lower than that observed in MDA-MB-231 cells (Fig. S4). Although counterintuitive, expression levels of integrins are not always a direct readout of integrin functions in cells [34]. Additional levels of regulation exist. Upon binding of integrins to extracellular matrix proteins, there is a crosstalk between integrins that determines downstream signaling and cell behavior [34]. For example, α5β1 and αvβ3 integrins both bind fibronectin and this is the collaborative interactions among these two integrins rather than their respective expression levels that determine cell migration response toward fibronectin [42]. Interestingly, the knockdown of integrin β1 in murine 4T1 triple-negative breast cancer cells induces a compensatory increase in β3 integrin expression and a switch in the migratory behavior of 4T1 cells from collective to single cell movement in vitro that leads to metastasis in vivo [43]. The β1 integrin subunit heterodimerizes with different α subunits [34], which probably explains why ITGA5 silencing did not modify cell surface expression levels of β1 and αvβ3 in MDA-MB-231 and MDA-B02 cells (Figs. S4 and S5). Yet, it is conceivable that α5β1 helps triple-negative breast cancer cells survive in the bone marrow until environmental conditions are sufficiently permissive for tumor growth, at which time integrin switching from α5β1 to αvβ3 triggers pro-invasive signals. This hypothesis warrants further investigation.

Having shown that there is an explicit role for ITGA5 in mediating early breast cancer cell colonization in the bone marrow, we then investigated whether ITGA5 also plays a role in the development of metastatic skeletal lesions. In bone metastasis, there is a vicious cycle where tumor cells stimulate osteoclast-mediated bone resorption and, in turn, bone-derived growth factors released from resorbed bone stimulate skeletal tumor burden [2]. We showed that the silencing or pharmacological inhibition of ITGA5 markedly reduced tumor outgrowth in experimental models of bone metastasis or tumorigenesis. This inhibition of tumor growth (and subsequent overall decrease in the secretion of tumor-derived pro-osteoclastic factors) led to the reduction in osteoclast-mediated bone destruction as would be predicted by the vicious cycle theory. However, there is some evidence in the literature showing that human osteoblasts and osteoclasts do express ITGA5 [35, 44]. It is possible that M200 could act upon human osteoblast differentiation. Here, we found that a clinically relevant concentration of M200 antibody was nearly as effective as the anti-RANKL antibody denosumab to inhibit human osteoclast differentiation and activity in vitro. Thus, in addition to its antitumor effect, we anticipate antibody M200 may also be effective at inhibiting bone resorption.

Clinical trials have repeatedly failed to demonstrate therapeutic benefits of integrin inhibitors in cancer patients [34]. Volociximab has shown preliminary evidence of efficacy in early phase I/II trials but failed in larger phase III trials [18, 20, 21]. However, none of these clinical trials using patients with advanced cancer and metastasis have specifically addressed the efficacy of volociximab on bone metastasis. A focus for further work would be to establish if ITGA5-positive breast cancer patients with bone metastases are likely to benefit from volociximab in combination with denosumab, which is the best standard of care for prevention of the skeletal morbidity associated with bone metastases in patients with advanced malignancies [2].

## Material and methods

### Patients

*ITGA5* mRNA expression was quantified by RT-qPCR in primary breast carcinomas obtained from the Curie Institute/René Huguenin Hospital (Saint-Cloud, France) [28]. ITGA5 protein immunohistochemistry was performed using primary tumors from breast cancer patients for whom the presence or absence of DTCs in bone marrow aspirates was known (University Medical Center Hamburg-Eppendorf, Germany) [29, 30, 45, 46].

### Analysis of human breast tumor microarray data sets

Analysis were conducted using public breast cancer microarray data sets GSE2034, GSE12276, GSE2603, and NKI295, consisting of 855 patients with clinical outcomes [47], and data sets GSE11078 and GSE14020 for 80 distant breast cancer metastases [48, 49].

### Real-time qPCR

PCR experiments were conducted as previously described [50]. All primers are shown in Table S3.

### Tissue microarray and immunohistochemistry

Immunodetection of ITGA5 in breast tumor tissue microarrays was performed following a previously described method [4].

### Cell lines and cell transduction

Human breast cancer cell lines T47D, MCF-7, Hs587T, SKBR3, BT-474, and MDA-MB-231 were obtained from the American Type Culture Collection (Manassas, VA) and authenticated using short tandem repeat analysis. The human MDA-B02 breast cancer cell line (MDA-B02) is a subpopulation of the MDA-MB-231 cell line (MDA-MB-231) that was selected for its high and selective efficiency to metastasize to bone in mice [33, 51].

Stable silencing of ITGA5 was achieved in luciferase2-expressing MDA-MB-231 and MDA-BO2 cells (MDA-231-shITGA5 and MDA-BO2-shITGA5, respectively) by transduction with lentiviral plasmids containing hairpin shRNAs targeting ITGA5. *ITGA5* was overexpressed in luciferase2-expressing MCF-7 cells (MCF-7luc2 ITGA5) using the amphotropic retroviral packaging system (Clontech).

### Cell-based assays

Tumor cell functions were investigated using cell adhesion and migration assays, and cell-cycle analysis, as previously described [40, 52, 53]. The effect of antibody M200 on differentiation of mature osteoclasts or osteoblasts was studied using previously described methods [36, 40, 54].

### Animal studies

All procedures involving animals, including housing and care, method of euthanasia, and experimental protocols were conducted in accordance with a code of practice established by the local ethical committee (Comité d’Expérimentation Animale de l’Université Claude Bernard Lyon 1, CEEA-55) under project licence MESR Number: APAFIS#4798-2016040510106615. Four-week-old female BALB/c nude mice were purchased from Janvier Laboratories (Saint-Berthevin). For bone metastasis experiments, immunodeficient BALB/c female *nude* mice were randomly assigned to receive intraperitoneal injection of M200 antibody or control IgG (15 mg/kg) 1 day before tumor cell injection. MDA-BO2 cells were then inoculated into the tail artery (5.10^5^/100 μL of PBS) of anesthetized mice at day 0. Alternatively, MDA-BO2 shCtrl or MDA-BO2 shITGA5 cells (5.10^5^/100 μL of PBS) were inoculated into the tail artery. Hormone-responsive MCF-7luc2 ITGA5 and MCF-7luc2 control cells (4 × 10^5^ cells/100 μL of PBS) were injected intra-arterially to female BALB/c *nude* mice 2 days after subcutaneous implantation of 17β-estradiol pellet in animals. Measurements of the extent of osteolytic lesions and skeletal tumor burden were performed by radiography/microcomputed tomography and bioluminescence imaging, respectively, as previously described [40, 51–53]. On day 28 after tumor cell inoculation, anesthetized mice were sacrificed by cervical dislocation, and hind limbs were collected and embedded in paraffin for further analyses by histomorphometry, histology, and immunohistochemistry. Ex vivo bone marrow micrometastasis and tumorigenesis experiments were conducted as previously described [40, 51–53].

### Statistical analysis

All statistical analyses were performed using PASW Statistics (version 21.0; SPSS Inc., Chicago) or GraphPad Prism (version 5, San Diego) [55]. All in vitro experimental procedures consisted of at least three independent biological repeats, and appropriate negative and positive controls. Comparisons were performed using two-sided unpaired Student’s *t* test or ANOVA test followed by Tukey’s test for in vitro experiments and by Mann–Whitney *U* test for in vivo experiments with significance at <0.05 being used to determine significant differences. Cox proportional regression model was used to estimate hazard ratios and 95% CIs for distant relapse to bone in relation to the ITGA5 expression as a continuous variable, with adjustment for classic prognostic factors: age, tumor size, node involvement, estrogen receptor and progesterone receptor status, and Her2 status. Survival analyses were visualized using Kaplan–Meier plots and differences in survival across the strata were calculated using a log-rank *p*-test. In total, data were obtained from 855 patients with, as the first site of relapse, bone (*n* = 238), brain (*n* = 49), lung (*n* = 101), or liver metastasis (*n* = 107). For analyses of bone relapse free survival, non-bone events, including liver, lung, and brain relapses were censored.

The power calculation for ex vivo experiments is based on our previous work [40, 52, 53], showing that bone marrow micrometastases occur in 80% of animals. Similarly, for bone metastasis experiments, 80% of animals have skeletal lesions 4 weeks after intra-arterial tumor cell inoculation [17, 40, 51–53]. Assuming a power of 80% and a level of significance of 5%, we estimated that we will be able to measure a difference of 60% or greater with ten animals per group, using a Mann–Whitney test. With regard to tumor xenograft experiments, the tumor take in this animal model is 90–100%. From our previous work [17, 40, 51–53], assuming a power of 80% and a level of significance of 5%, we estimated that we will be able to measure a difference of 60% or greater with five animals per group, using a Mann–Whitney test. Only the animals that were alive at the end of the protocols were included in the statistical analyses.

In order to avoid bias for in vivo experiments, staff injecting transduced tumor cells into animals were different from those assessing the effects of transduction. Mice and subsequent tissue samples were labeled such that staff assessing the effects of transduction and analyzing the results were unaware which group received mock-transduced tumor cells or tumor cells in which ITGA5 was silenced or overexpressed until analyses were complete.

### Additional methods

A more detailed description of methods outlined above and additional methods used in this study are provided in Supplementary Methods section.

## Supplementary information

Figures S1 to S11

TABLE S1

TABLE S2

TABLE S3

Supplementary methods
