# Supplementary material for: Integrin alpha5 in human breast cancer is a mediator of bone metastasis and a therapeutic target for the treatment of osteolytic lesions
Source: Oncogene. 2021 Jan 8;40(7):1284–99. doi: 10.1038/s41388-020-01603-6 (PMC7892344; doi:10.1038/s41388-020-01603-6)
Supplement: Supplementary file 1 — Figures S1 to S11 [file 41388_2020_1603_MOESM1_ESM.pdf]

Supplementary Figures

Integrin alpha-5 in human breast cancer is a mediator of bone metastasis and a therapeutic target for the treatment of osteolytic lesions

Francesco Pantano, Martine Croset, Keltouma Driouch, Natalia Bednarz-Knoll, Michele Iuliani, Giulia Ribelli, Edith Bonnelye, Harriet Wikman, Sandra Geraci, Florian Bonin, Sonia Simonetti, Bruno Vincenzi, Saw See Hong, Sofia Sousa, Klaus Pantel, Giuseppe Tonini, Daniele Santini and Philippe Clézardin

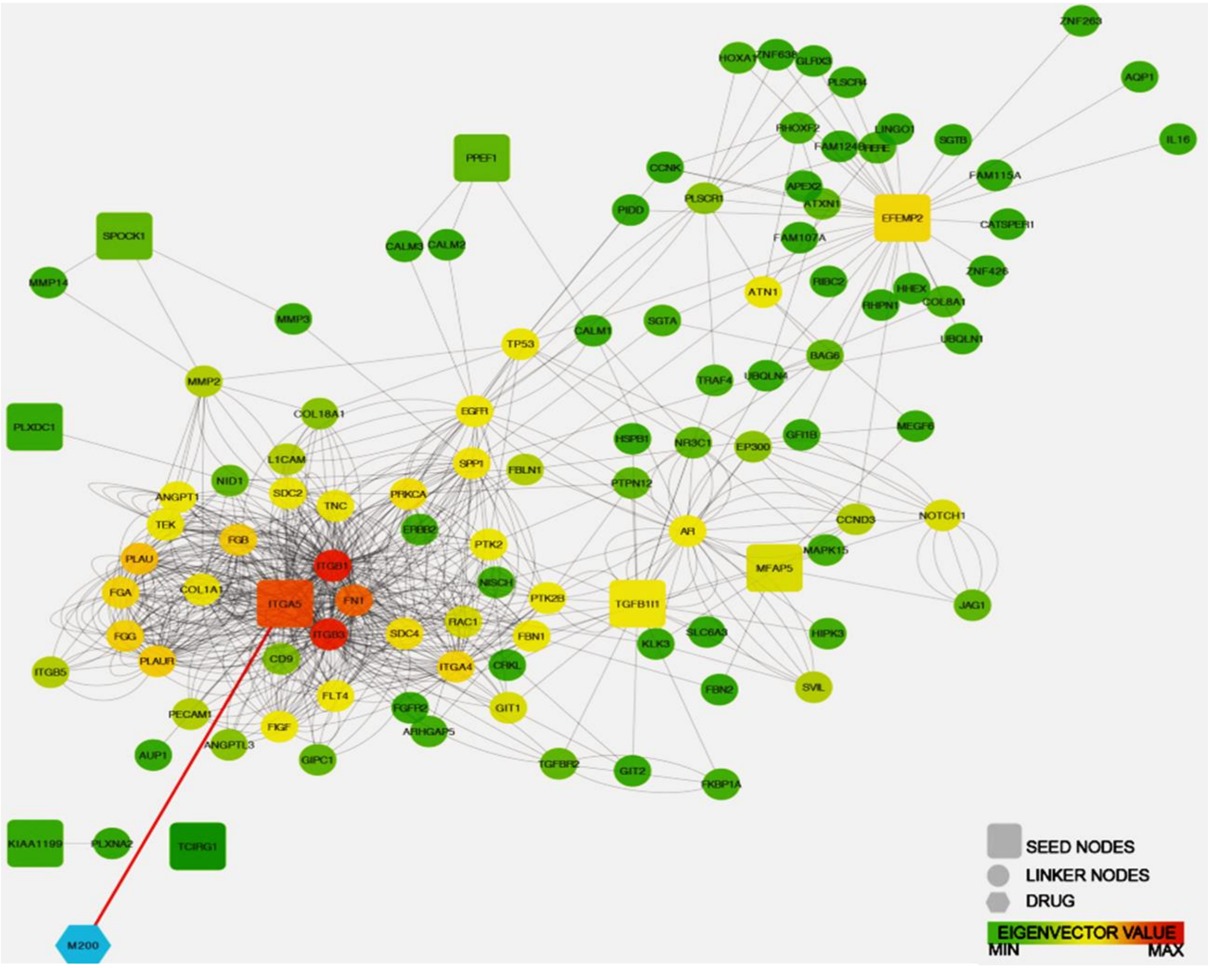

Figure S1: Gene interaction network analysis of 9 genes associated with bone metastasis. The network shows the nine genes (seed nodes) in the context of biological interactions derived from public pathway databases. Eigenvector value gives an immediate evaluation of the relative relevance of each node. ITGA5 had the highest eigenvector value among seed nodes. In addition, its companion integrin subunit ITGB1 and its ligand fibronectin (FN1) were among the highest eigenvector values as linker nodes.

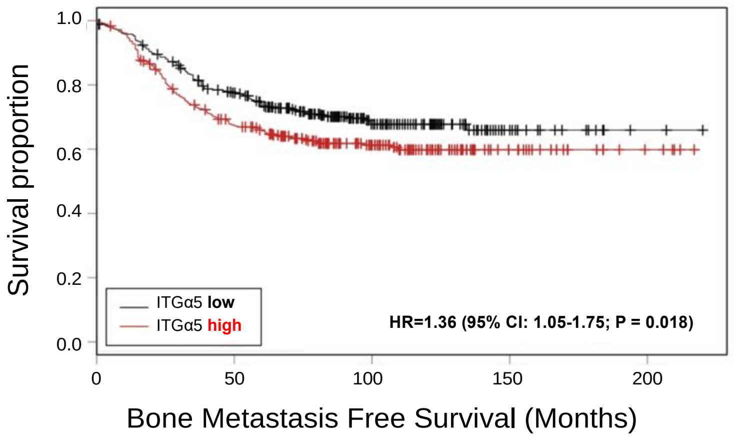

Multivariate Cox Regression analysis

	Hazard Ratio	95% C.I. Lower Limit	95% C.I. Upper Limit	p Value
**Age**	0.990	0.972	1.008	0.283
**Tumor Size**	1.011	0.996	1.025	0.141
**Nodal Status**	2.677	1.655	4.331	**0.000**
**Estrogen Receptor Status**	1.664	0.904	3.063	0.102
**Progesterone Receptor Status**	0.628	0.381	1.034	0.068
**Her2 Status**	1.209	0.730	2.000	0.461
**ITGA5 expression**	1.676	1.041	2.697	**0.034**

Abbreviations: C.I., confidence interval

Figure S2: *Upper panel:* Kaplan-Meier estimates for rates of bone metastasis-free survival of breast cancer patients (n = 855), according to *ITGA5* high and low expression levels in primary tumors (GSE2034, GSE12276, GSE2603 and NKI295). HRs and 95% CIs are based on Cox univariate analysis. *Lower panel*: Multivariate Cox regression analysis. HR: hazard ratio; CI: confident interval.

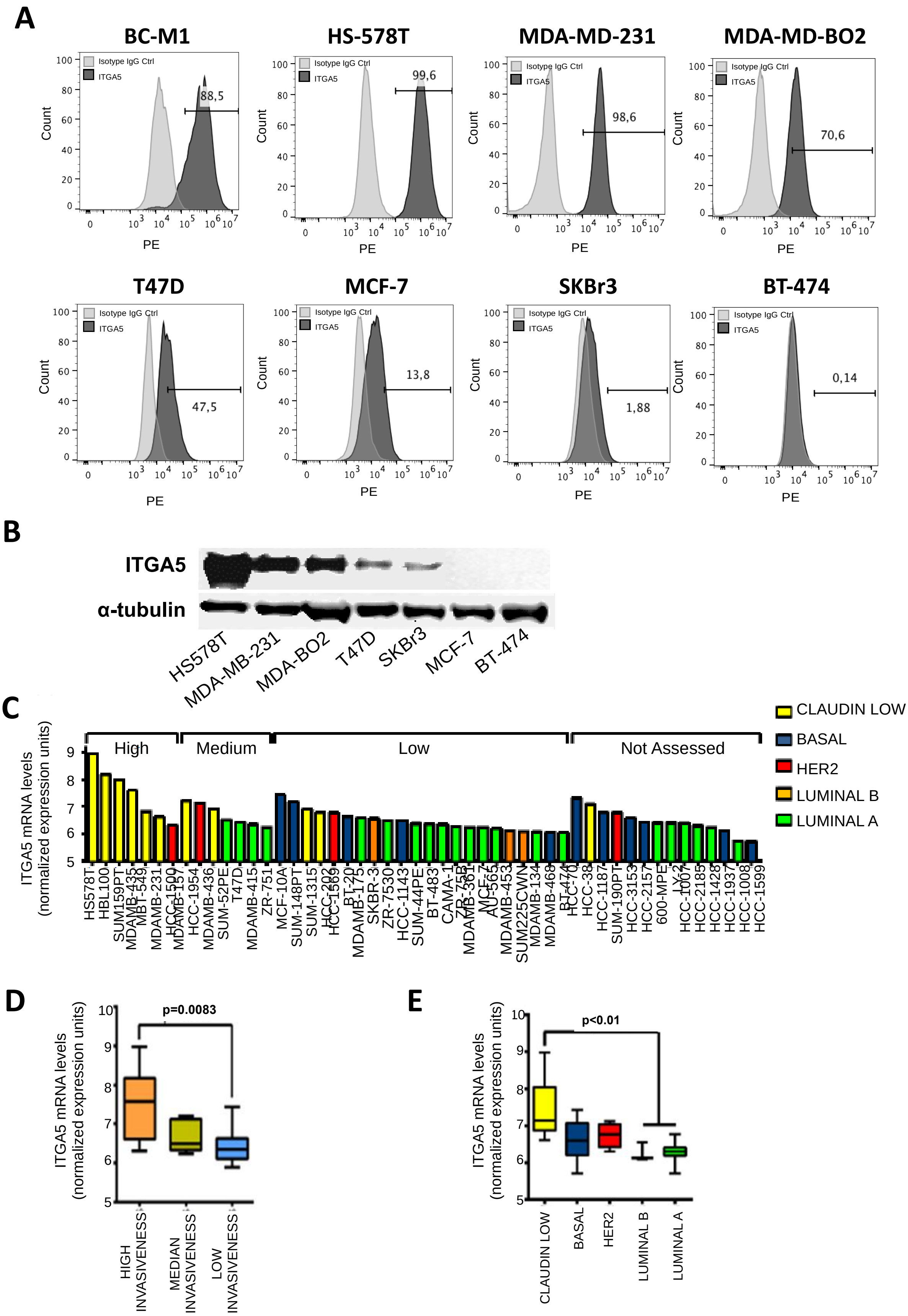

A
BC-M1
HS-578T
MDA-MD-231
MDA-MD-BO2
T47D
MCF-7
SKBr3
BT-474
Isotype IgG Ctrl
ITGA5
Count
PE
88,5
99,6
98,6
70,6
47,5
13,8
1,88
0,14
B
ITGA5
α-tubulin
HS578T
MDA-MB-231
MDA-BO2
T47D
SKBr3
MCF-7
BT-474
C
ITGA5 mRNA levels (normalized expression units)
High
Medium
Low
Not Assessed
CLAUDIN LOW
BASAL
HER2
LUMINAL B
LUMINAL A
D
ITGA5 mRNA levels (normalized expression units)
p=0.0083
HIGH INVASIVENESS
MEDIAN INVASIVENESS
LOW INVASIVENESS
E
ITGA5 mRNA levels (normalized expression units)
p<0.01
CLAUDIN LOW
BASAL
HER2
LUMINAL B
LUMINAL A

Figure S3: ITGA5 expression in human breast cancer cell lines. **(A)** Cell surface expression levels of integrin α5β1 in triple-negative (BC-M1, Hs578t, MDA-MB-231, MDA-B02), luminal A (T47D, MCF-7, BT-474) and HER2-expressing luminal B (SKBr3) human breast cancer cell lines as measured by flow cytometry using anti-ITGA5 monoclonal antibody IIA1 (black histograms) or an isotype-matched negative control antibody (grey histograms). (**B)** Western blot analysis of ITGA5 protein expression in human Hs-578T, MDA-MB-231, MDA-BO2, T47D, MCF-7, SKBr3 and BT-474 breast cancer cell lines**. (C)** *ITGA5* mRNA expression level plotted according to the molecular phenotype and degree of invasiveness of 51 human breast cancer cell lines (GSE12777). **(D)** Correlation of *ITGA5* mRNA levels with invasive/metastatic capacities of human breast cancer cell lines. **(E)** Same as (D) for molecular breast cancer subtypes.

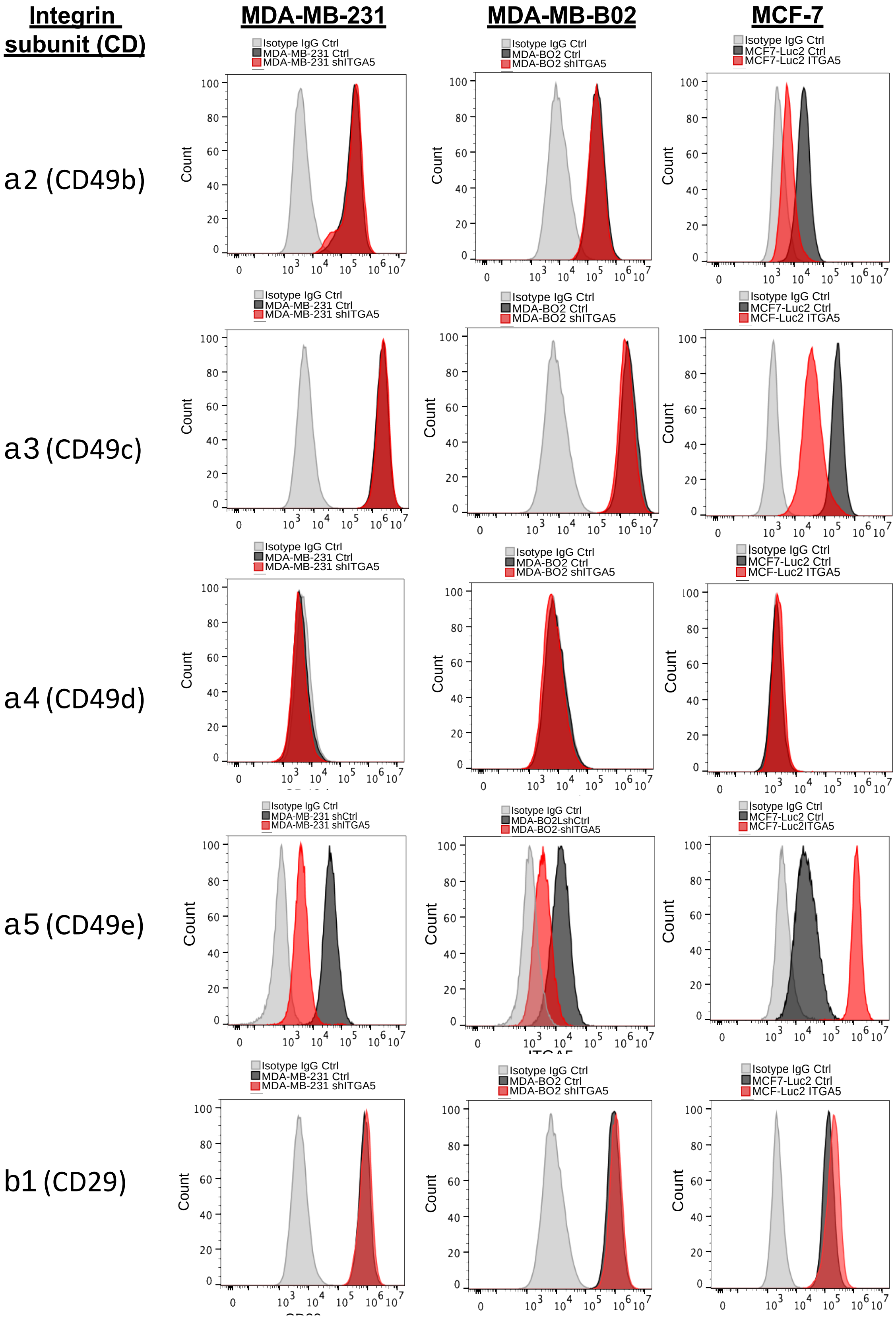

Figure S4: Cell surface expression levels of integrins α2, α3, α4, α5 and β1 in human MDA-MB-231, MDA-B02 and MCF-7 breast cancer cell lines. MDA-MB-231 and MDA-B02 cells were transduced with a plasmid containing a shRNA targeting ITGA5 (shITGA5; red histograms) or

a non-targeting shRNA (Ctrl; black histograms). MCF-7 cells were transfected with a vector containing ITGA5 (red histograms) or an empty vector (Ctrl; black histograms). Nonspecific binding of antibodies was evaluated using an isotype-matched antibody and Ctrl-breast cancer cell lines (grey histograms). CD: cluster of differentiation.

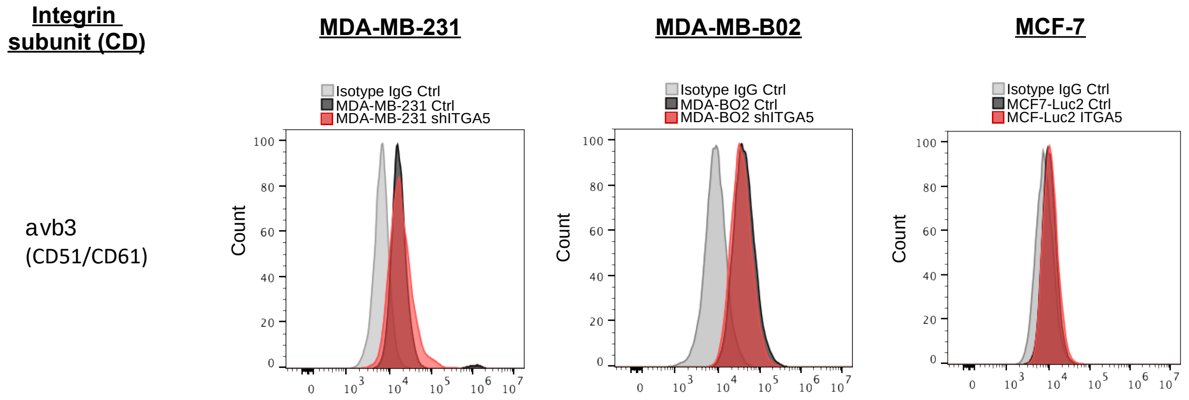

Figure S5: Cell surface expression levels of integrin αvβ3 in human MDA-MB-231, MDA-B02 and MCF-7 breast cancer cell lines. MDA-MB-231 and MDA-B02 cells were transduced with a plasmid containing a shRNA targeting ITGA5 (shITGA5; red histograms) or a non-targeting shRNA (Ctrl; black histograms). MCF-7 cells were transfected with a vector containing ITGA5 (red histograms) or an empty vector (Ctrl; black histograms). Nonspecific binding of antibodies was evaluated using an isotype-matched antibody and Ctrl-breast cancer cell lines (grey histograms). CD: cluster of differentiation.

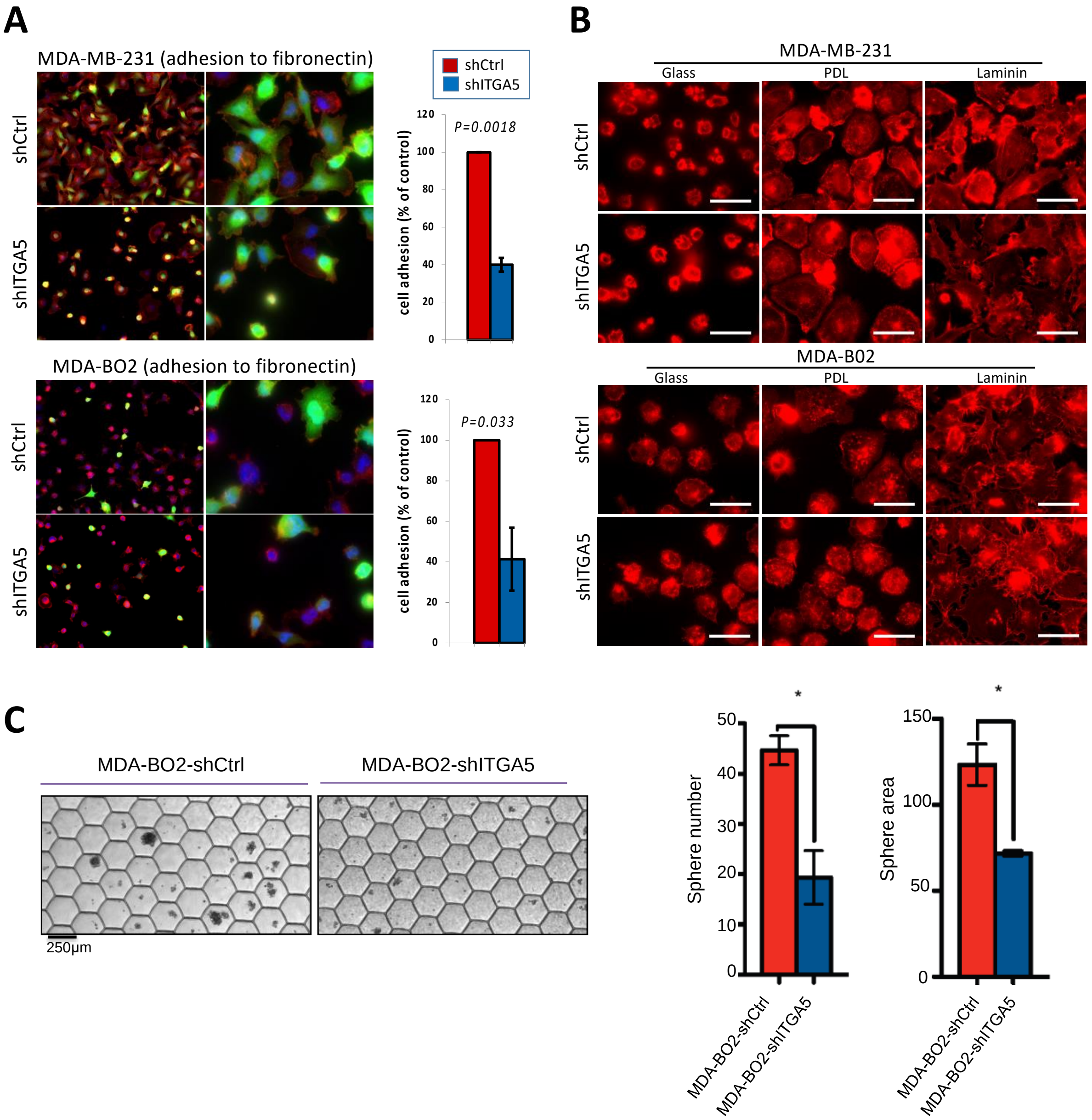

Figure S6: ITGA5 silencing in breast cancer cells impairs cell adhesion to fibronectin and the formation of tumor spheroids *in vitro* and reduces the progression of osteolytic lesions *in vivo*. **(A)** Representative images of MDA-MB-231 or MDA-BO2 cell adhesion to fibronectin. MDA-MB-231 and MDA-BO2 cell silenced or not for ITGA5 were seeded on coverslips pre-coated with fibronectin and incubated for 1 hour at 37°C prior to fixation. Cells were stained with DAPI to visualize the nuclei and with rhodamine-labelled phalloidin to visualize F-actin (red) whereas EGFP (green) localized to cytosol is given by the transduction plasmid. Images were acquired under epifluorescence microscopy (left panels: X10 and right panels: X40). Bar graphs represent the % of shITGA5 cells attached to fibronectin relative to shCtrl cells (set to 100%).

(B) Representative images of MDA-MB-231 and MDA-BO2 shITGA5 and shCtrl cells attached to glass, poly-D-lysine (PDL) or laminin after a 1-hour incubation at 37°C (magnification: x40). (**C**) Representative images and analysis of MDA-BO2-shCtrl or MDA-BO2-shITGA5 cells grown as mammospheres in ultra-low adherence microchambers, under serum-free medium. The ITGA5 silencing induced a significant decrease in the number and size of mammospheres. Data are mean ± SEM.*: $P < 0.05$.

A

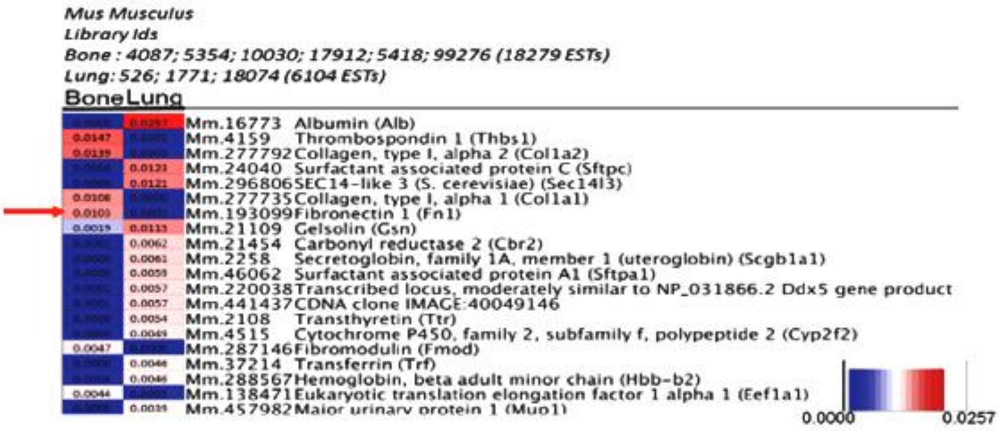

B

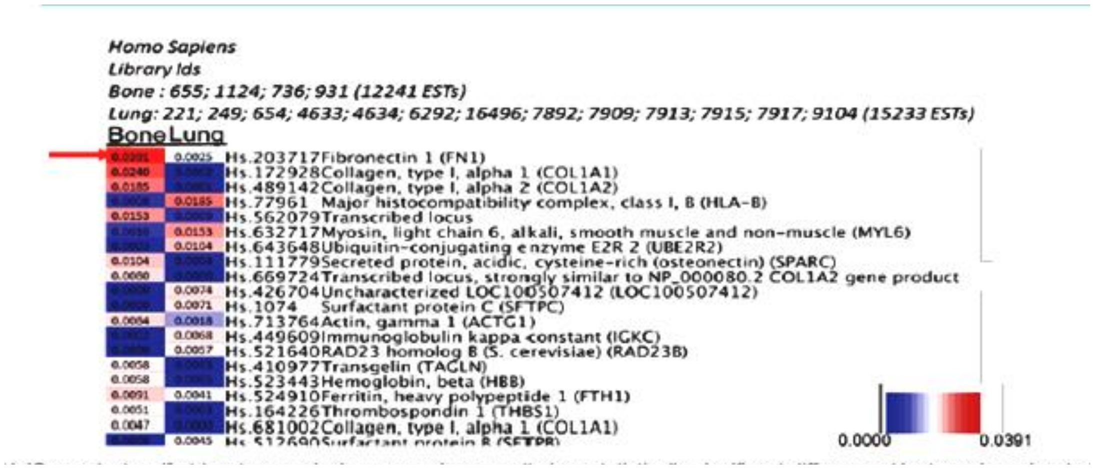

Figure S7: Bone marrow stroma and lung parenchyma EST (expressed sequence tag) profiles analysis in *Mus Musculus* (**A**) and *Homo Sapiens* tissue samples (**B**).

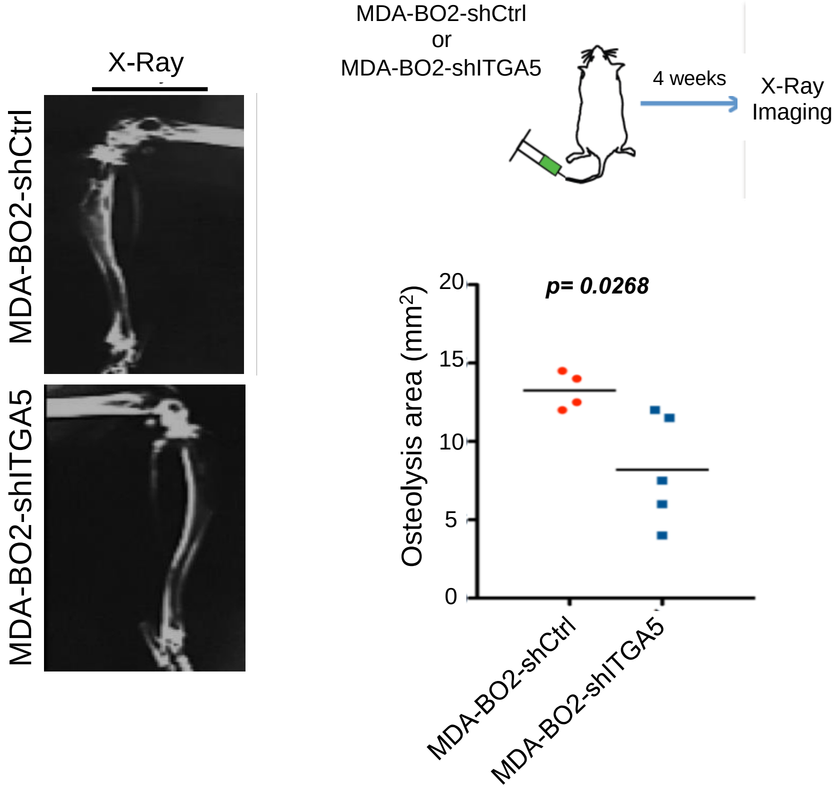

Figure S8: Schematic representation of the experimental protocol. MDA-B02-shCtrl or MDA-B02-shITGA5 cells were inoculated intra-arterially to Balb/c *nude* mice (n = 4 to 5 per group). Four weeks after tumor cell inoculation, animals were analyzed by radiography (X-Ray). *Left panel*: Representative radiographs of metastatic legs for each group 4 weeks after tumor cell inoculation. *Right panel*: Quantification of the area of osteolytic lesions (mm^2) on radiographs 4 weeks after tumor cell inoculation.

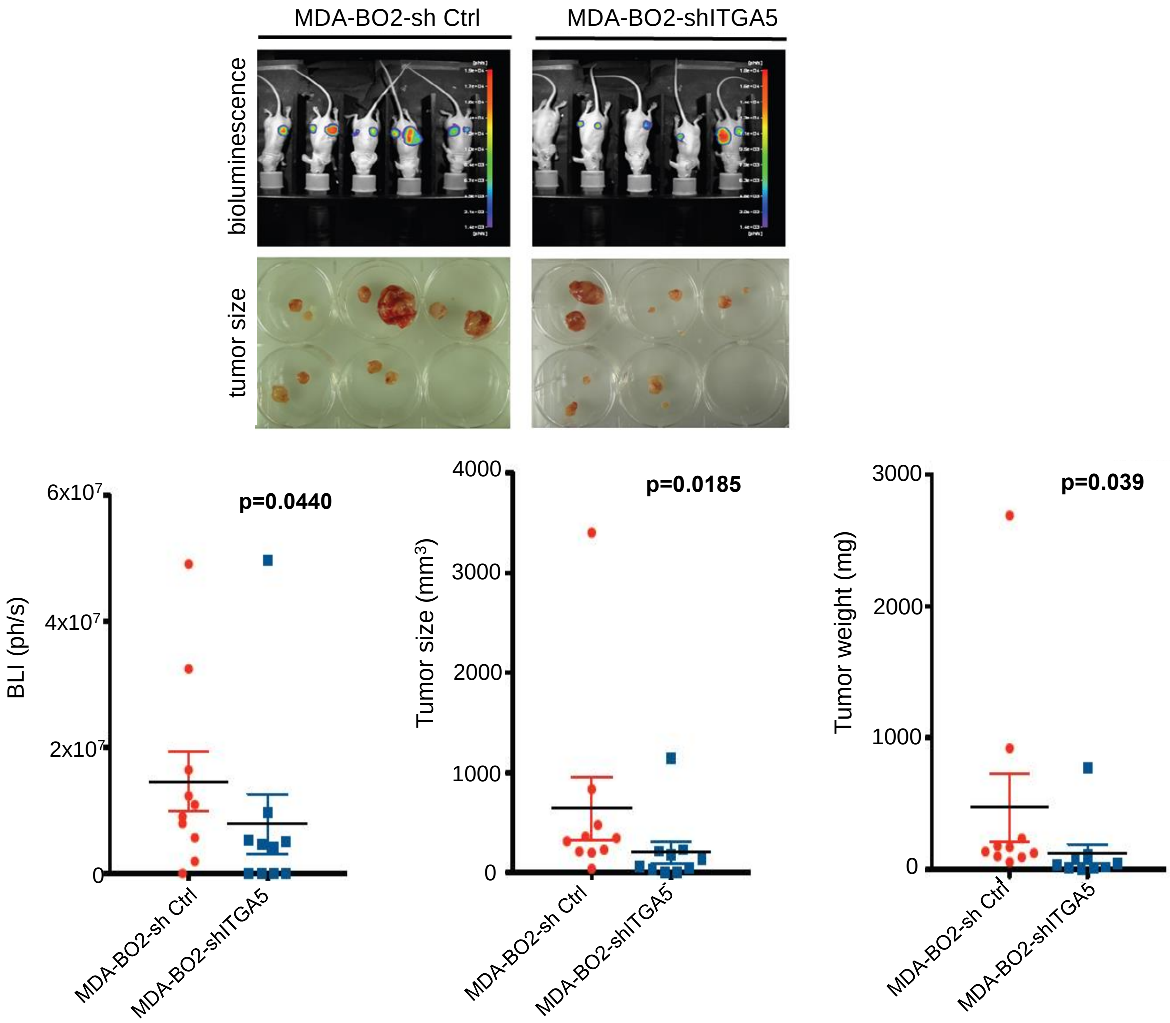

Figure S9: Genetic inhibition of ITGA5 reduces subcutaneous growth of MDA-B02 breast tumors *in vivo*. Tumorigenesis experiments were conducted using MDA-B02 breast cancer cells silenced for ITGA5 (MDA-BO2 shITGA5) and control MDA-B02 cells (MDA-BO2 shCtrl) that were injected subcutaneously (right and left flank) to Balb/c *nude* mice. At day 60 post-injection, animals were culled and tumors weighted. *Top panel*: whole-body bioluminescence imaging (BLI) and photographs of tumors for each group (5 animals per group). *Bottom panel*: Differences in tumor growth between the two groups at day 60, as assessed by BLI, measurement with a Vernier caliper and tumor weighting.

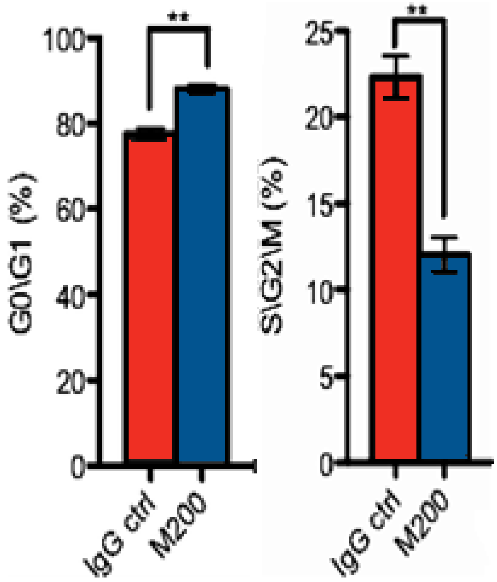

Figure S10: MDA-MB-231/B02 cells were synchronized overnight by serum starvation, incubated with 250 µg/ml of humanized anti α5 monoclonal antibody (M200) or a control human IgG and then seeded to fibronectin precoated chambers under serum-free condition. Cell cycle analysis was performed 72 hours after staining with propidium iodide. Data are mean ± SEM. Statistical significance was determined using unpaired Student t test; ** significance $p<0.001$

A

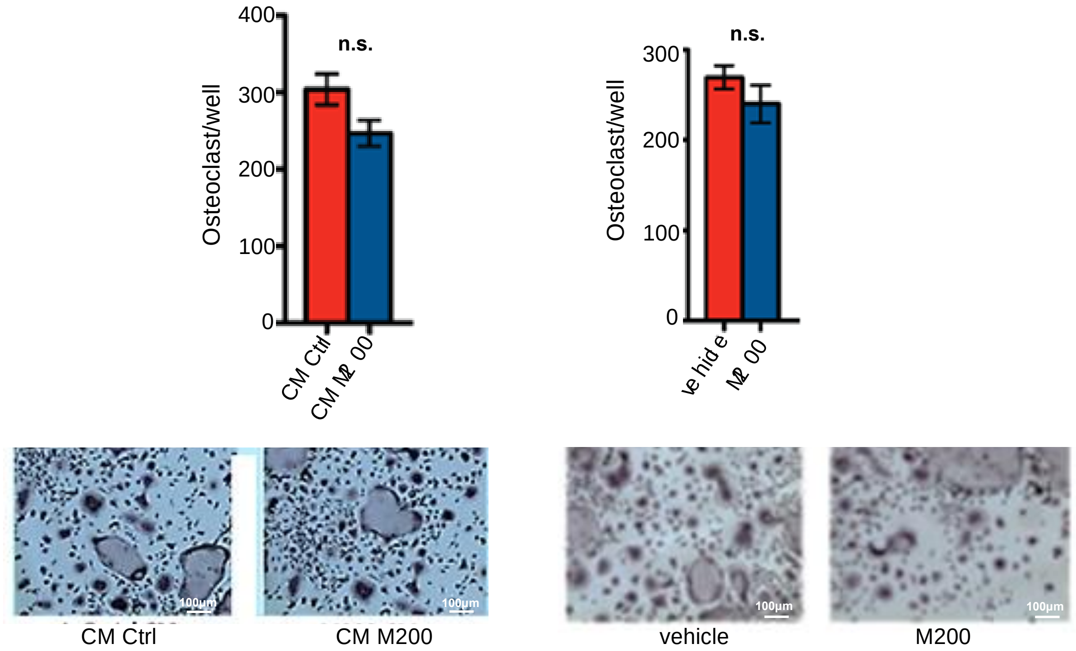

B

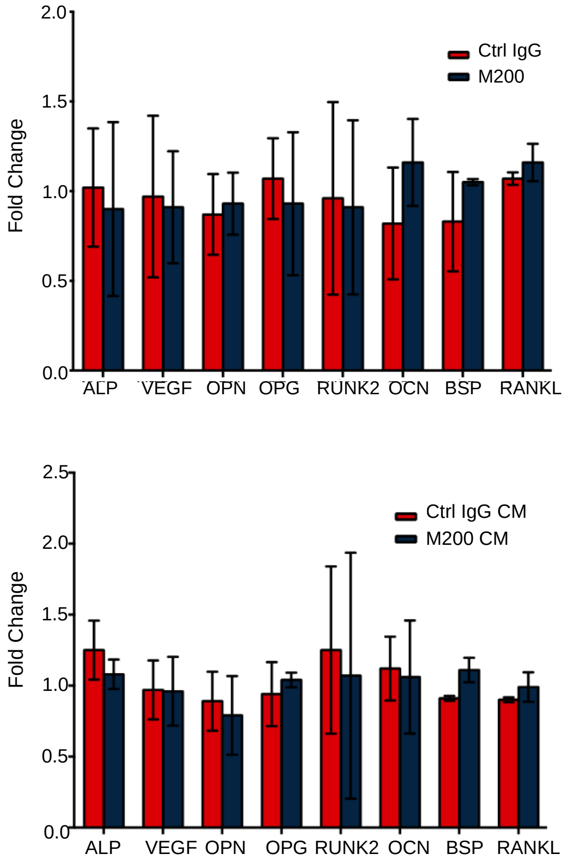

Figure S11: M200 does not inhibit murine osteoclastogenesis and osteoblastogenesis *in vitro*.

(A) *Left panel:* Effect of the conditioned medium (CM) from MDA-B02 breast cancer cells

treated with M200 (250 µg/ml) or a control IgG (250 µg/ml) on murine osteoclastogenesis. *Right panel*: Effect of M200 treatment on murine osteoclastogenesis, compared to vehicle. **(B)** *Upper panel*: Effect of M200 treatment (250 µg/ml) on murine MC3T3-E1 cell differentiation, compared to vehicle, as measured by mRNA expression levels of alkaline phosphatase (ALP), osteopontin (OPN), bone sialoprotein (BSP), osteocalcin (OCN), RUNX2, VEGFA, osteoprotegerin (OPG) and RANKL. *Lower panel*: Effect of the conditioned medium (CM) from MDA-B02 breast cancer cells treated with M200 (250 µg/ml) or a control IgG (250 µg/ml) on murine MC3T3-E1 cell differentiation.
