# Supplementary material for: Integrin alpha5 in human breast cancer is a mediator of bone metastasis and a therapeutic target for the treatment of osteolytic lesions
Source: Oncogene. 2021 Jan 8;40(7):1284–99. doi: 10.1038/s41388-020-01603-6 (PMC7892344; doi:10.1038/s41388-020-01603-6)
Supplement: Supplementary file 3 — TABLE S2 [file 41388_2020_1603_MOESM3_ESM.docx]

**Table S2.** Association between ITGA5 protein expression in radically resected primary tumors, as assessed by immunohistochemistry, and clinical and biological characteristics of 268 breast cancer patients with or without DTCs in the bone marrow.

| **Characteristics** | | **ITGA5 expression** | | | | **Chi-2 test**  **(p-value)** |
| --- | --- | --- | --- | --- | --- | --- |
|  |  | **Negative**  **n** | **%** | **Positive**  **n** | **%** |  |
| Age (years) | <median  ≥median | 56  49 | 53.3  46.7 | 88  74 | 54.3  45.7 | 0.874 |
| Tumor status | T1  T2  T3  T4 | 51  41  6  7 | 48.6  39.0  5.7  6.7 | 90  61  8  3 | 55.6  37.7  4.9  1.9 | 0.200 |
| Node Status | N0  N1-3 | 60  44 | 57.7  42.3 | 97  65 | 59.9  40.1 | 0.724 |
| Grade score | G1  G2  G3 | 8  57  35 | 8.0  57.0  35.0 | 9  92  59 | 5.6  57.5  36.9 | 0.743 |
| Bone marrow  status | negative  positive | 84  18 | 82.4  17.6 | 113  46 | 71.1  28.9 | **0.039** |
| Molecular Subtype | HR-pos  TNBC  HER2-pos | 72  16  2 | 80  17.8  2.2 | 111  23  10 | 77.1  16.0  6.9 | 0.276 |
| Ki-67 expression cut-off: 10% | negative  positive | 42  41 | 50.6  49.4 | 68  82 | 45.3  54.7 | 0.440 |
