# Supplementary material for: Integrin alpha5 in human breast cancer is a mediator of bone metastasis and a therapeutic target for the treatment of osteolytic lesions
Source: Oncogene. 2021 Jan 8;40(7):1284–99. doi: 10.1038/s41388-020-01603-6 (PMC7892344; doi:10.1038/s41388-020-01603-6)
Supplement: Supplementary file 4 — TABLE S3 [file 41388_2020_1603_MOESM4_ESM.docx]

**Table S3.** Primers used for plasmid construct and RT-qPCR gene expression analysis
