# Supplementary material for: Integrin alpha5 in human breast cancer is a mediator of bone metastasis and a therapeutic target for the treatment of osteolytic lesions
Source: Oncogene. 2021 Jan 8;40(7):1284–99. doi: 10.1038/s41388-020-01603-6 (PMC7892344; doi:10.1038/s41388-020-01603-6)
Supplement: Supplementary file 5 — Supplementary methods [file 41388_2020_1603_MOESM5_ESM.docx]

**Supplementary Methods**

**Patients**

For the cohort of breast cancer patients (n = 427) from the Curie Institute/René Huguenin Hospital (Saint-Cloud, France) (29), the median follow-up was 120.5 months (range = 13–347 months). Among these patients, 92 of them developed bone metastases. For the breast cancer cohort from the University Medical Center Hamburg-Eppendorf (Germany), radically resected primary breast tumors (n = 268) collected from 1999 to 2006. Bone marrow status was assessed for all patients. The patients from all cohorts received endocrine treatment, radiotherapy and/or chemotherapy according to the best standard of care. The variable clinical, pathological and molecular parameters were documented. TNM staging was applied according to the American Joint Committee on Cancer 7th edition recommendations, whereas molecular subtype classification was assigned as previously described (46).

**Analysis of human breast tumor microarray data sets**

Four public breast cancer microarray data sets (GSE2034, GSE12276, GSE2603 and NKI295) consisting of 855 patients with clinical outcomes, including the first site of distant relapse, were combined to perform survival analyses, as described by Harrell *et al*. (47)_._

Transcriptomic data of skin, lung, liver, brain and bone metastases from breast cancer patients (n=80) obtained from the Curie Institute/René Huguenin Hospital (GSE11078) (Saint-Cloud, France), the University of L’Aquila (L’Aquila, Italy) and IDIBELL (Barcelona, Spain) (GSE14020) were included (48-50). Differential Expression Analysis was performed with Gene Pattern suite (<https://genepattern.broadinstitute.org>).

A class comparison was conducted based on univariate t-test applying filtering criteria (*P* < 10^-4^, fold-change>1.5) for genes that are not highly expressed in normal bone, as compared to normal breast.

**Tissue microarray immunohistochemistry**

Each TMA comprised of 0.6 or 1.5 mm-diameter tissue cores obtained from formalin-fixed paraffin embedded breast cancer specimens. Fragments of normal breast, colon, lung, prostate and endometrium were introduced to TMAs as internal controls. Sections were cut 4-6 µm thick and placed on charged polylysine-coated slides (Superfrost Plus, BDH). ITGA5 staining was performed on deparaffinized TMA sections treated for 5 min in citrate buffer pH 6.0 (Biogenex) at 120°C in a steamer and then incubated for 16 hours at 4°C with monoclonal anti-α5β1 antibody (HA5, MAB1969, Chemicon, Millipore) (1/500, dilution in REALTM Antibody Diluent; Dako). The immunostaining was envisioned by ChemMate Detection Kit Peroxidase/DAB, Rabbit/Mouse (Dako) and slides were counterstained with hematoxylin. The immunostaining intensity of ITGA5 protein expression in tumor cells was scored as negative, weak, moderate or strong. Subcellular localization of the staining and percentage of positive tumor cells were documented. For semi-quantitative analysis, the staining intensity was multiplied by the percentage of stained cells. The optimal cut-off point was evaluated by the use of the receiver operating characteristic method.

**Cell culture and transduction**

Human breast cancer cell lines T47D, MCF-7, Hs587T, SKBR3, BT-474, and MDA-MB-231 were obtained from the American Type Culture Collection (ATCC, Manassas, VA, USA). These cell lines and the human MDA-B02 breast cancer cell line were cultured according to standard protocols (52). BC-M1 is a DTC tumor cell line obtained from a bone marrow aspirate of a breast cancer patient with no clinical sign of distant metastasis at the time of primary tumor resection (32). Cell cultures were routinely tested for mycoplasma by measuring the luminescence intensity from cell culture supernatant using the MycoAlert™ PLUS Mycoplasma Detection Kit (Lonza, the Netherlands). Breast cancer cell lines were transduced to express luciferase2 using the pantropic retroviral expression packaging system (Clontech Laboratories), as described (41).

Stable silencing of ITGA5 was achieved in luciferase-2-expressing MDA-MB-231 and MDA-BO2 cells (MDA-231-shITGA5 and MDA-BO2-shITGA5, respectively) by transduction with lentiviral plasmids containing hairpin shRNAs targeting ITGA5. MDA-MB-231 and MDA-BO2 cells transduced with the universal non-targeting shRNA (pZIP-hCMV-UltramiRshRNA lentiviral vector, Transomic Technologies) were used as negative control cell lines (MDA-MB-231-shCtrl and MDA-BO2-shCtrl, respectively). shRNA was designed by the shERWOOD algorithm (Dr. G.Hannon Cold Spring Harbor Laboratory) and cloned into the UltramiR scaffolds for optimal shRNA processing, according to the manufacturer. Lentiviral particles were obtained by transfecting the Lenti-X 293T cell line with the Lenti-X HTX Packaging System and the pZIP-hCMV-shRNA plasmid in the presence of Xfect Transfection Reagent, according to the manufacturers’ guidelines (Takara, Clontech Laboratories). Tumor cells were incubated with lentiviral particles in presence of RetroNectin reagent and cultured in complete medium containing tetracycline-free fetal bovine serum (FBS) (Takara) and then subjected to antibiotic selection with 2 μg/mL puromycin.

For ITGA5 overexpression in MCF-7 cells, the GP2-293 cell line expressing the viral GAG and POL genes was first transfected with both the pantropic VSV-G vector and the bicistronic retroviral expression vector pQCXIP (Clontech). The ITGA5 coding sequence was PCR-amplified from the pCB-7-ITGA5 plasmid (Dr Giancotti FG, MSK cancer center, NY, Addgene) and inserted into the pQCXIP vector by homologous recombination using In-Fusion HD Cloning kit (Clontech). Tumor cells were incubated with the retroviral particles in the presence of 8 μg/ml polybrene prior to selection with 2 μg/ml puromycin.

**Real-Time qPCR**

Total RNA was isolated using a standard acid-phenol guanidium method. RNA concentration and purity were measured on Nanodrop ND-1000 (Nanodrop Technologies, Wilmington, DE). TATA box–binding protein (TBP) transcripts were used as an endogenous RNA control. First-strand cDNA was synthesized using a SuperScriptII Reverse-Transcriptase kit (Invitrogen), according to the manufacturer’s guidelines. Real-time quantitative PCR (RT-qPCR) was performed with the SYBR Green PCR Core Reagents kit (Applied Biosystems) on an ABI Prism 7700 Sequence Detection System (Applied Biosystems) and gene expression in each sample was normalized on the basis of TBP expression. Standard curves were performed using a serial dilution of cDNA pool consisting of several normal tissue-specific TBP and ITGA5 primers. The linearity of standard curves was verified, with all coefficients of variation between 0.96 and 0.99.

For human breast cancer cells, total RNA was extracted using the Nucleospin RNA kit (Macherey Nagel, Duren). cDNA was produced from 1 µg RNA using the iScript cDNA Synthesis Kit (Bio-Rad). RT- qPCR reactions were performed using a SYBR Green qPCR kit (Invitrogen). Relative gene expression levels were normalized according to the Ct value of the gene encoding the ribosomal protein L32 and results were expressed as fold differences equal to 2^-ΔΔCt^ (41). Human osteoclast gene expression was analyzed by Taqman RT-qPCR after RNA extraction with Trizol. All primers are shown in **Supplementary Table S3**.

**Digital Differential Display**

Digital Differential Display, a bioinformatic tool available at the National Center for Biotechnology Information ([www.ncbi.nlm.nih.gov/UniGene/info_ddd](http://www.ncbi.nlm.nih.gov/UniGene/info_ddd).html), analyzes the frequencies of cDNA and expressed sequence tag (EST) in expression libraries. This technique compares the constituent sequences of different libraries to determine the relative frequency of each transcript in the libraries analyzed. Fisher exact test is used to determine the statistical significance of the number of times that sequences from the selected libraries are assigned to a specific UniGene cluster. In this manner, the relative abundance of transcripts in each library is determined and thus differential gene expression of genes can be identified. In particular the following library Ids were included in the analysis: 5418, 9927 (*Mus Musculus* bone marrow); 526, 1771, 18074 (*Mus Musculus* Lung); 736, 931, 17382 (*Homo Sapiens* bone marrow); 221, 249, 654, 16406, 18357, 18363, 18521 (*Homo Sapiens* Lung).

**Western blot, immunofluorescence and FACS**

For western blotting, tumor cells in culture were harvested and lysed in RIPA buffer (Sigma) containing a protease cocktail inhibitor (Roche) and protein extracts electrophoresed on a 4-12% gradient SDS-polyacrylamide gel (Thermofisher scientific), then transferred to nitrocellulose membranes. For osteoclast protein analysis, cells were lysed in RIPA buffer containing proteinase inhibitors and protein extracts (20 μg of total proteins) were electrophoresed on 4%-15% gradient SDS-PAGE gels, transferred onto nitrocellulose membranes through Trans-Blot Turbo Transfer System (Bio-Rad) and incubated with a blocking buffer (TBST 1X with 5% non-fat dry milk) for one hour. Electrophoresed tumor proteins that were transferred onto membranes were probed with primary antibodies against ITGA5 (Rabbit polyclonal antibody AB1949, Chemicon, Millipore, Bedford, MA), or β-tubulin (Cl B-512, Sigma), according to manufacturers’ instructions. For transferred osteoclast proteins, membranes were incubated for 2 hours at room temperature with mouse monoclonal anti-human CD49e (610633, BD Biosciences) and mouse anti-human actin-β (Sigma-Aldrich) antibodies. Membranes were then incubated with horseradish peroxide (HRP)-conjugated donkey anti-rabbit and anti-mouse secondary antibodies (Amersham) prior to immunostaining with enhanced chemiluminescence (ECL) detection system (Perkin Elmer) or ChemiDoc (Bio-Rad). Quantity One software (Bio-Rad) was used to quantify the band signal intensity.

Tumor cell surface expression analysis of integrins by flow cytometry was performed by incubating tumor cells (2x10^5^ cells in 100μl PBS-1% BSA) with primary antibody against ITGA2 (clone AK-7, BD Biosciences), ITGA3 (clone C3 II.1, BD Biosciences), ITGA4 (clone 9F10, BD Biosciences), ITGA5 (clone IIA1, BD Biosciences), integrin β1 (clone MAR4, BD Biosciences), αvβ3 (clone 23C6, BD Biosciences) or α5β1 (clone HA5, MAB1999, Chemicon, Millipore) or using an IgG isotype control antibody (mouse IgG1, IgG2b; BD Biosciences). For flow cytometry analysis, cultured tumor cells were harvested with trypsin/EDTA, except for αvβ3 cell surface expression analysis where cells were harvested with EDTA only.

Immunofluorescence study was conducted with osteoclasts grown at 70–80% confluency on glass coverslips (Carlo Erba) and fixed using 4% (v/v) paraformaldehyde for 20 min at 37°C. Permeabilization was performed with 0.1% Triton X-100 for 10 min at room temperature. After incubation for 30 min with 10% (w/v) bovine serum albumin to block nonspecific binding, cells were incubated with mouse PE anti-human CD49e antibody (clone IIA1) (BD Biosciences) and Phalloidin-FITC for 1 hour at 37°C. Coverslips were counterstained with 2 μg/mL 4,6-diamidino-2 phenylindole (DAPI) (SigmaAldrich) in anti-fade Vectashield (Vector Laboratories). Images of each sample were acquired using a Nikon Ti fluorescence microscope. For human breast cancer cell lines, immunofluorescence study was conducted with tumor cells seeded on glass coverslips pre-coated with matrix (3X10^4^ cells in 1 ml medium) and placed in 6-well plates. Cells were incubated at 37°C prior to fixation and permeabilization for 10 min with PBS containing 4% (v/v) paraformaldehyde and 0.1% (v/v) Triton. Cells were then incubated with PBS containing 1.5% bovine serum albumin (w/v) and stained for 10 min at 37°C with PBS containing 0.5 μM tetramethylrhodamine-Phalloidine (Sigma) and 1 μg/ml DAPI (diamidino-2-phenylindole) to visualize the nuclei. Coverslips were mounted using FluoroSave reagent (Calbiochem), imaged using epifluorescence microscopy (Axiovert 200M) and computerized with ImageJ.

**M200 antibody**

M200 (Volociximab, Eos 200-4) is a high-affinity IgG4 chimeric (82% human, 18% murine) monoclonal antibody that specifically binds to human form of ITGA5 without any specific binding to the murine form of ITGA5 (18). M200 was supplied in 150 mg (20mL) single-use vials by PDL Biopharma, Inc, USA. The composition of each vial was 10 mg/mL M200, 25 mmol/L citrate, 150 mmol/L sodium chloride and 0.05% polysorbate (Tween-80, pH 6.5).

**Tumor Cell-based functional assays**

Cell adhesion assays were performed in 24-well plates coated with type-I collagen, vitronectin or fibronectin matrix (10 μg/ml). MDA-BO2 cells (10^5^ cells per wells) were pre-incubated with M200 antibody or control human IgG and cells were then allowed to adhere for 1h on the matrix in the absence of serum. Cells were fixed and stained with crystal violet and counted under microscope. Alternatively, 1 hour after plating, MDA-MB-231 and MDA-BO2 cell adhesion and spreading to type-I collagen (5μg/cm^2^), vitronectin (10μg/cm^2^), laminin (1μg/cm^2^) and fibronectin (1μg/cm^2^) was studied by immunofluorescence after paxillin labelling in order to enumerate adherent cells and visualize cell spreading. The adherence of MCF-7luc2 cells on matrix was also studied by quantification of bioluminescence signals. Briefly, MCF-7luc2 Ctrl and MCF-7-luc2 ITGA5 cells were resuspended in culture medium without FBS (10^5^ cells per well) and seeded in 24-well culture plates pre-coated or not pre-coated with matrix proteins. After 30-min incubation at 37°C, adherent cells were rinsed with PBS and lysed with 100-μl lysis buffer to measure bioluminescence signals using the Luciferase Assay System (Promega) on a Luminoscan Ascent luminometer (Thermo Electron).

Cell migration assays were conducted in 24-well cell culture plates with 8-µm diameter pore-size inserts pre-coated with fibronectin (100 µl; 10 μg/ml), as previously described (41,53,54). For anchorage-independent tumor spheroid (mammosphere) formation assays, MDA-BO2 cells were seeded in CytoCapture chambers (Big hexagonal cavities, diameter 250μm) (Mobitec, Germany) and grown in suspension in 0.3mL of MammoCult^TM^ basal medium containing 10% (v/v) MammoCult^TM^ proliferation supplement (Stem Cell), 1μg/mL hydrocortisone, 1mM glutamine, 50U/mL penicillin and 50μg/mL streptomycin. Tumor spheroids were imaged under microscope (Confocal-Leica SP5 X) and quantified using Fiji software. Spheroids of at least 70μm in diameter were counted.

Cell cycle analysis was performed by fluorescence analysis after tumor cell staining by propidium iodide (BD cycle test Plus DNA Reagent kit, BD Biosciences).

**Osteoclastogenesis assays**

Bone marrow cells from 6-week-old OF1 male mice were cultured for 7 days in differentiation α-MEM medium containing 10% (v/v) fetal calf serum (Invitrogen), 20 ng/mL of macrophage colony-stimulating factors (M-CSF; R&D Systems), and 50 ng/mL of soluble recombinant receptor activator of nuclear factor κB ligand (RANKL; R&D Systems) (37,41). Cells were continuously (day 1–7) exposed to the conditioned medium from MDA-BO2 cells (25 μg proteins for each condition). After 7 days in culture, mature multinucleated osteoclasts were stained for TRAP activity (Sigma-Aldrich) and counted as osteoclast when containing 3 or more nuclei.

Primary human osteoclasts were differentiated from human peripheral blood mononuclear cells (PBMCs) of female healthy blood donors as previously described (37). Specifically, PBMCs were isolated with Lympholyte®-H density gradient (Cedarlane Laboratories) and monocytes were sorted using anti-human CD14 antibody with MoFlo Astrios Cell Sorter (Beckman Coulter). Isolated monocytes were then cultured for 12 days in RPMI culture medium (Euroclone) supplemented with 10% (v/v) fetal bovine serum (Euroclone), 100 units/ml penicillin, 100 mg/ml streptomycin (Euroclone), 2 mM L-glutamine (Euroclone), 25 ng/mL M-CSF and 50 ng/mL RANKL (R&D Systems) in order to induce osteoclast differentiation. At day 12, culture medium was removed, cells were fixed with 4% (v/v) formaldehyde for 5 minutes and stained with leukocyte acid phosphatase (TRAP) kit (Sigma-Aldrich) according to the manufacturer's instructions. TRAP-positive cells (>3 nuclei) were then counted. Osteoclast activity was assessed by culturing cells on plates coated with a synthetic inorganic bone mimetic matrix (Osteoassay, Corning). At day 12, the culture medium was removed and plates filled with sodium hypochlorite solution to evaluate the ability of mature osteoclasts to reabsorb this substrate. The pits produced by osteoclasts were quantified by ImageJ software. M200 (250 μg/ml), denosumab (250 μg/ml) or an IgG Ctrl antibody was added to the culture medium starting from day 0 until the end of the differentiation protocol, and the culture medium was changed every 3 days. Osteoclast viability was assessed at the end of the osteoclast differentiation protocol using a MTT assay (Sigma Aldrich), as described in the manufacturer’s instructions.

**Analysis of human osteoclast gene expression**

Total RNA was extracted from osteoclast using the Trizol reagent (Invitrogen) according to the manufacturer’s instructions. cDNA was produced using the High Capacity cDNA Reverse Transcription kit (Applied Biosystems) according to the manufacturer’s instructions. mRNA levels were measured by quantitative real-time polymerase chain reaction (qRT-PCR) using TaqMan Gene Expression Assays (**Supplementary** **Table S3**) in 7900HT Real- Time PCR System (Applied Biosystems). Gene expression levels were normalized to the endogenous housekeeping gene glucuronidase beta (GUSβ).

**Osteoblastogenesis assay**

MC3T3-E1 were seeded in 12-well plates (10^5^ cells/well) and cultured in αMEM medium containing 10% (w/v) FBS, supplemented with 50 μg/mL ascorbic acid at day 1 and 10 mM β-glycerophosphate at day 8 in order to induce differentiation over 21 days (60). Conditioned media from tumor cells were prepared as described above and incubated (25 μg/mL protein) with osteoblastic cells two days after plating and changing medium every two days, until the end of experiments. After checking for Von Kossa mineralization with one well, remaining wells were washed with PBS and cells were harvested in lysis buffer for RNA extraction and qRT-qPCR analysis, as previously described (41,55).

**Animal studies**

For bone metastasis experiments, radiographs of metastatic immunodeficient BALB/c female *nude* mice (LifeRay HM Plus, Ferrania) were taken using a MX-20 cabinet X-ray system (Faxitron X-ray Corporation, Wheeling). The area of osteolytic lesions identified on radiographs was measured using a computerized image analysis system (MorphoExpert-Exploranova). The extent of bone destruction was expressed in square millimeters, as previously described (41). The progression of osteolytic lesions in hind limbs of animals was also monitored by micro-computed tomography (µCT), using the scanner SkyScan1176 (Bruker). For µCT analysis, acquisition settings were 9-μm voxel size and an X-ray tube (50 kV; 500μA, 1000ms exposure time) with a 0.5-mm aluminum filter. Three-dimensional reconstructions, rendering and quantitative analyses were performed using the manufacturer’s guidelines (respectively, NRecon, CTVox, and CTAn). The BV/TV and bone mineral density (BMD) measurements included trabecular and cortical bone and were performed in a defined region of interest (ROI) of the tibiae. The ROI was contained between 0.5 and 1.5 mm below the growth plate, used here as the morphological reference for positioning the ROI. For measurement of skeletal tumor burden, bioluminescence imaging of animals was performed weekly using the Nightowl imaging system (Berthold).

For e*x vivo* bone marrow micrometastasis experiments (53,54), tumor cells (5 x 10^5^/100 μL PBS) were injected into the tail artery of BALB/c *nude* mice. And animals were then culled on day 7 after tumor cell inoculation. Hind limbs were collected and tibiae and femurs were minced and incubated for 2 hours at 37°C in DMEM medium containing 300U/ml type-I collagenase and 100 U/ml hyaluronidase (StemCell Technologies). Minced lungs were incubated in buffer containing 0.25 mg/ml type-I collagenase (Sigma) for 1 hour at 37°C. Cell suspensions from bone marrow and lungs were seeded in 6-well plates. Cultured cells were then placed under puromycin selection for 2 weeks, allowing the selective growth of antibiotic-resistant tumor cells. Colonies of tumor cells were then fixed and stained with crystal violet.

For tumor xenograft experiments, MDA-BO2 transfectants were inoculated subcutaneously (10^6^ cells/100 μL PBS) to female BALB/c *nude* mice (52-54). Tumor size was calculated by external measurement of the width (m_1_) and length (m_2_) of subcutaneous tumors using a Vernier caliper. Tumor volume (TV) was calculated using the equation TV = (m_1_^2^×m_2_)/2. At the end of the protocols, mice were culled and tumors collected.

**Bone histomorphometry and histology**

Hind limbs from animals were fixed and embedded in paraffin. Five-micrometer sections were stained with Goldner's Trichrome and processed for histomorphometric analysis to calculate the bone volume/tissue volume (BV/TV) and the tumor burden/soft tissue volume (TB/STV) ratio. The *in situ* detection of osteoclasts was carried out on metastatic bone tissue sections using the tartrate-resistant acid phosphatase (TRAP) activity Kit assay (Sigma-Aldrich). The ratio of TRAP-stained, osteoclast-active bone resorption surfaces (Oc.S) to the total bone surface (BS) was calculated using the computerized image analysis system MorphoExpert (Exploranova).

**Immunohistochemistry**

Five-μm paraffin sections of lungs and decalcified long bones were incubated overnight with a fibronectin antibody (1/100)(bs-0666R, Bioss antibodies) followed by incubation with horseradish peroxidase (HRP)-conjugated anti-rabbit (Dako) antibody for 1 hour and the signal was then developed with 3,3’-diaminobenzidine (Dako). Tissue sections were counterstained with Mayer’s hematoxylin (Merck) and mounted. For KI-67 staining, 5-μm paraffin-embedded bone tissue sections were incubated with a mouse anti-human Ki-67 monoclonal antibody (DakoCytomation) and the mitotic index was quantified as described (41,52-54).

**Additional Statistical analyses**

To identify genes correlating with bone metastasis-free survival, a Cox model analysis was performed on the normalized gene expression data with a FDR-adjusted p-value threshold of 0.05 using Pomelo II suite (<http://pomelo2.iib.uam.es>).

Analysis for Eigenvector centrality was performed using Centiscape 1.0 plugin for Cytoscape 2.8. on binary or pairwise interaction network consisting of interactions derived from HPRD, NCI-Nature Pathway Interaction Database, Reactome, and the MSKCC Cancer Cell Map. The network was constructed with current data updates in Pathway Commons, as described (56).

The dichotomous cutoff value to categorize breast cancer patients for low and high ITGA5 mRNA expression was derived using the receiver operating characteristic method. To test the effect of ITGA5 expression on clinical outcome, survival distributions were estimated using the Kaplan-Meier method, and the significance of differences between survival rates was ascertained using the log-rank test. Multivariable analysis using Cox proportional hazards model was used to assess the independent contribution of each variable to bone metastasis free survival. Finally, a chi square test was used to determine any significant association between clinical pathological variables and ITGA5 expression. All statistical tests were two-sided. P values less than 0.05 were considered to be statistically significant and, where appropriate, the difference in means and the 95% confidence intervals (95% CIs) were generated.
